# Combination finerenone and empagliflozin for chronic kidney disease with type 2 diabetes: a target trial emulation

**DOI:** 10.3389/fneph.2026.1930977

**Published:** 2026-10-20

**Authors:** Lei Li, Dan Zhang, Manrong Xu, Shaohua Li, Yun Shen, Shengdi Lu

**Affiliations:** 1 Department of Nephrology, Deyang Hospital affiliated to Chengdu University of Traditional Chinese Medicine, Deyang, China; 2 Department of Endocrinology, Shanghai Sixth People’s Hospital Affiliated to Shanghai Jiao Tong University School of Medicine, Shanghai, China; 3 Department of Nephrology, Southwest Hospital affiliated to Army Medical University, Chongqing, China; 4 Pennington Biomedical Research Center, Baton Rouge, LA, United States

**Keywords:** diabetic kidney disease, empagliflozin, finerenone, real-world evidence, target trial emulation

## Abstract

**Objective:**

This study aims to emulate the CONFIDENCE trial and compare combination finerenone plus empagliflozin with each monotherapy for albuminuria reduction in Chinese patients with chronic kidney disease (CKD) and type 2 diabetes (T2D).

**Design:**

This study employed modified target trial emulation. The primary contrast was the per-protocol effect, estimated by cloning, censoring, and inverse probability of censoring weighting, which is not susceptible to grace-period immortal time bias; a propensity-score-matched assignment analysis, the observational analogue of intention-to-treat, was secondary.

**Setting:**

Electronic health records from three Chinese tertiary (Grade A) hospitals, from January 2023 to September 2025, were utilized.

**Participants:**

A total of 2,895 adults (965 per strategy) with CKD and T2D met the CONFIDENCE eligibility criteria, selected by 1:1:1 propensity score matching from 3,822 eligible patients.

**Main outcome measures:**

Relative change in urine albumin-to-creatinine ratio (UACR) from baseline to day 180 was the main outcome measure (co-primary comparisons with Holm–Bonferroni correction).

**Results:**

At day 180, the median UACR reduction was 52.0% in the combination strategy, 32.1% with finerenone, and 28.9% with empagliflozin. In the per-protocol analysis the difference was −19.5 percentage points (95% confidence interval −20.5 to −18.5) versus finerenone and −22.8 (−23.8 to −21.8) versus empagliflozin. The secondary assignment analysis gave −19.6 (−20.5 to −18.7) and −22.9 (−23.8 to −22.0), respectively (both *P* < 0.001); this is not a true intention-to-treat effect and is supportive only. Hyperkalemia occurred in 12.7%, 12.8%, and 3.5%, respectively, acute kidney injury in 4.9%, 2.8%, and 2.3%, respectively, and symptomatic hypotension in 1.9%, 0.6%, and 0.4%, respectively. The findings were consistent across 10 sensitivity analyses and six subgroups.

**Conclusions:**

Combination finerenone and empagliflozin was associated with greater albuminuria reduction than either monotherapy, directionally consistent with CONFIDENCE. Because the emulated strategy permitted sequential initiation within 90 days whereas CONFIDENCE randomized simultaneous initiation, this is a modified emulation rather than a direct replication. Albuminuria is a surrogate endpoint and follow-up was 180 days, so hard kidney and cardiovascular outcomes remain unstudied. Despite loss to follow-up of 29.6% to 32.7%, the finding was robust in a tipping-point analysis. Hyperkalemia was frequent with finerenone, so biochemical monitoring remains necessary. Findings apply to comparable Chinese tertiary-care populations.

## Highlights

• What was known:

- Finerenone and sodium-glucose cotransporter 2 inhibitors independently reduce albuminuria and slow chronic kidney disease progression in patients with type 2 diabetes.
- The CONFIDENCE trial demonstrated that the combination of finerenone and empagliflozin produces additive albuminuria reduction compared with either monotherapy.
- No data existed on the effectiveness or safety of this combination in Chinese patients, who differ from the predominantly Western CONFIDENCE population in age, body mass index, and prescribing patterns.

• This study adds:

- In the per-protocol clone-censor-weight analysis, which is not susceptible to immortal time bias from the grace period, combination therapy reduced the urine albumin-to-creatinine ratio at day 180 by 19.5 percentage points more than finerenone and 22.8 percentage points more than empagliflozin, directionally consistent with CONFIDENCE. The emulated strategy permits sequential initiation and is therefore broader than the simultaneous initiation tested in CONFIDENCE.
- Hyperkalemia occurred in 12.7%–12.8% of finerenone-containing arms versus 3.5% with empagliflozin alone; acute kidney injury and symptomatic hypotension were numerically more frequent with combination therapy.
- Only the short-term albuminuria surrogate was replicated; hard kidney and cardiovascular endpoints were not assessed within the 180-day follow-up, and substantial loss to follow-up that could not be evaluated under a missing-not-at-random assumption further limits certainty.

• Potential impact:

- These findings provide the first multicenter real-world evidence on finerenone–empagliflozin combination therapy in Chinese tertiary care and may inform forthcoming updates to the Chinese expert consensus on finerenone.
- The target trial emulation framework demonstrated here offers a principled approach for extending randomized trial evidence to under-represented populations using routine clinical data.
- Potassium monitoring remains essential in finerenone-containing regimens regardless of concomitant empagliflozin use, even in populations with lower observed hyperkalemia rates.

## Introduction

Diabetic kidney disease (DKD) is the leading cause of end-stage kidney disease worldwide (1). In China, the prevalence of CKD among adults exceeds 10%, with diabetes mellitus as the fastest-growing etiology (2). Despite renin–angiotensin system (RAS) blockade with angiotensin-converting enzyme inhibitors (ACEi) or angiotensin receptor blockers (ARB), many patients continue to experience progressive albuminuria, declining kidney function, and cardiovascular events (3). This residual risk has driven the search for additional therapeutic strategies targeting complementary pathological pathways.

Two drug classes have reshaped the management of CKD with T2D in recent years. SGLT2 inhibitors reduce kidney and cardiovascular events through hemodynamic, metabolic, and anti-inflammatory mechanisms, as demonstrated in the CREDENCE, DAPA-CKD, and EMPA-KIDNEY trials (4–6). Independently, finerenone, a nonsteroidal mineralocorticoid receptor antagonist (nsMRA), reduces albuminuria and slows CKD progression through anti-fibrotic and anti-inflammatory effects, as established in the FIDELIO-DKD and FIGARO-DKD trials (7, 8) and their pooled FIDELITY analysis (3). Because these two classes act through distinct mechanisms, their combination holds theoretical promise for additive cardiorenal protection.

The CONFIDENCE trial tested this hypothesis directly (9). In 800 adults with CKD and T2D, the combination of finerenone plus empagliflozin produced a 52% reduction in UACR at day 180, compared with 32% for finerenone alone and 29% for empagliflozin alone. Albuminuria is an accepted surrogate endpoint that predicts, but does not establish, long-term cardiorenal benefit (10, 11). However, CONFIDENCE was a phase 2 trial powered for a surrogate endpoint. It enrolled a predominantly Western population (mean age, 68 years) with a limited 180-day follow-up. No data exist on the effectiveness of this combination in Chinese patients, whose clinical profile differs substantially in terms of younger age, lower body mass index (BMI), overwhelmingly ARB-based RAS inhibition, and a higher stroke-to-myocardial infarction ratio (12). Published Chinese real-world experience with finerenone remains limited to small, single-center studies (13, 14).

Target trial emulation (TTE) provides a principled framework for estimating causal treatment effects from observational data by explicitly mapping the protocol of a hypothetical randomized controlled trial (RCT) onto real-world data sources (15, 16). When applied rigorously, TTE avoids common biases in observational research, including immortal time bias, prevalent user bias, and confounding by indication (17). This approach has been successfully applied to comparative effectiveness questions in diabetes (18) and is well suited for extending RCT findings to populations not represented in the original trial.

We emulated the CONFIDENCE trial using electronic health record (EHR) data from three Chinese tertiary hospitals. Our objectives were to estimate the per-protocol effect of combination finerenone plus empagliflozin versus each monotherapy on UACR change at day 180 as the primary causal contrast, to report the corresponding assignment contrast, the observational analogue of an intention-to-treat (ITT) comparison, as a secondary contrast, and to compare the safety profile of the combination in a Chinese clinical setting. We reported this study in accordance with the TARGET statement (19).

## Methods

### Target trial specification

We conducted this retrospective study following the TTE framework (15, 16) and reported it in accordance with the TARGET statement (19). The protocol of the hypothetical target trial was modeled on the CONFIDENCE trial (9). **Table 1** presents the full specification of the target trial and describes how each protocol component was emulated using observational data. The causal assumptions underlying this emulation are depicted in **Supplementary Figure S1**. The study design, including the alignment of eligibility assessment, treatment assignment, and the start of follow-up at time zero, is illustrated in **Figure 1** (20).

**Table 1:** Summary of target trial emulation protocol.

Component	Target trial protocol	Emulation using Chinese EHR data
Eligibility criteria	Adults ≥18 years with T2D (HbA1c <11%) and CKD (eGFR 30–90 mL/min/1.73 m², UACR ≥100 to <5,000 mg/g), serum K^+^ ≤4.8 mmol/L, on stable maximally tolerated ACEi or ARB (≥1 month). Excluded: T1D, HFrEF (NYHA II–IV), cardiovascular events within 90 days, SGLT2i or MRA use within 8 weeks, K^+^ >4.8 mmol/L, prior kidney transplant, AKI within 6 months, non-diabetic CKD, severe liver disease	ICD-10 codes E11.x (T2D) and N18.x (CKD) identified in HIS. eGFR calculated using CKD-EPI 2021 equation from serum creatinine (μmol/L) in LIS. Spot urine UACR (mg/g) from LIS. Serum K^+^ from LIS (≤4.8 mmol/L). Stable ACEi/ARB verified via ≥2 consecutive outpatient prescription fills with no dose change in 30 days before T0. Exclusions applied using ICD-10 diagnosis codes and procedure codes from HIS inpatient/outpatient records with specified look-back windows (3 years for comorbidities, 90 days for CV events, 6 months for AKI, 60 days for SGLT2i/MRA washout)
Treatment strategies	Strategy A: finerenone (10/20 mg QD per Chinese label) + empagliflozin (10 mg QD). Strategy B: finerenone monotherapy (10/20 mg QD). Strategy C: empagliflozin monotherapy (10 mg QD). All strategies require background RAS inhibition (ACEi or ARB). Finerenone dose: 20 mg if eGFR ≥60, 10 mg if eGFR 25–<60; up-titration after 4 weeks if K^+^ ≤4.8 and eGFR decline <30%	Treatment strategies identified from outpatient pharmacy dispensing records using Chinese drug names. A 90-day grace period applied for combination strategy classification to accommodate sequential initiation common in Chinese clinical practice. Dose and titration captured from prescription records
Assignment procedures	Eligible individuals would be randomly assigned in a 1:1:1 ratio, stratified by eGFR (<60 vs. ≥60 mL/min/1.73 m²) and UACR (≤850 vs. >850 mg/g)	Patients classified into treatment strategies at time zero based on filled prescriptions. Propensity score matching (1:1:1) using a multinomial logistic regression model incorporating all baseline covariates. Matching performed using nearest-neighbor algorithm with caliper of 0.2 SD of the logit propensity score. Balance assessed via standardized mean differences (threshold <0.10)
Follow-up period	Starts at treatment assignment (day 1). Ends at the earliest of day 180, death, loss to follow-up, or administrative end of study	Starts at time zero (date of first qualifying prescription fill). Ends at earliest of 180 days, in-hospital death, last clinical encounter (>90-day gap = loss to follow-up), or September 30, 2025. Visit windows: day 30 (± 14 days), day 90 (± 21 days), and day 180 (± 30 days)
Outcomes	Co-primary: relative change in UACR from baseline to day 180, combination vs. finerenone alone, and combination vs. empagliflozin alone. Secondary: proportion achieving ≥30%/≥40%/≥50% UACR reduction; eGFR change at day 30 and 180. Safety: hyperkalemia (K^+^ >5.5 mmol/L), AKI (KDIGO criteria), symptomatic hypotension, all-cause mortality	Primary: spot UACR (mg/g) from LIS closest to day 180 within ±30-day window, expressed as ratio to baseline. Secondary and safety outcomes operationalized using LIS laboratory values and ICD-10 diagnosis codes from HIS. All-cause mortality limited to in-hospital deaths (no linked death registry available)
Causal contrasts	Primary contrast: per-protocol (PP) effect, the effect of initiating and sustaining the assigned strategy. Secondary contrast: effect of strategy assignment (observational analogue of the intention-to-treat effect). Both prespecified	Assignment (secondary): propensity-score weighted comparison at time zero. PP: clone-censor-weight approach; patients censored at protocol deviation (discontinuation >60 days, switching agents, adding second study drug); inverse probability of censoring weights (IPCW) applied to adjust for informative censoring, with time-varying confounders (K^+^, eGFR, SBP) updated at 30-day intervals
Statistical analysis	MMRM on log-transformed UACR with treatment, visit, treatment × visit interaction, baseline log-UACR, eGFR category. Holm–Bonferroni correction for co-primary comparisons. Multiple imputation for missing data. Logistic regression for categorical secondary outcomes	Assignment analysis (secondary): linear regression on the log-UACR ratio within the PS-matched cohort, with robust standard errors. Least-squares mean ratios with 95% CIs. Per-protocol (primary): clone-censor-weight analysis with IPCW-weighted regression and bootstrap 95% CIs (500 iterations). Sensitivity analyses: E-value for unmeasured confounding, complete-case analysis, varied grace period, and IPTW as an alternative to matching. Subgroup analyses by eGFR category and UACR category

**Figure 1:**
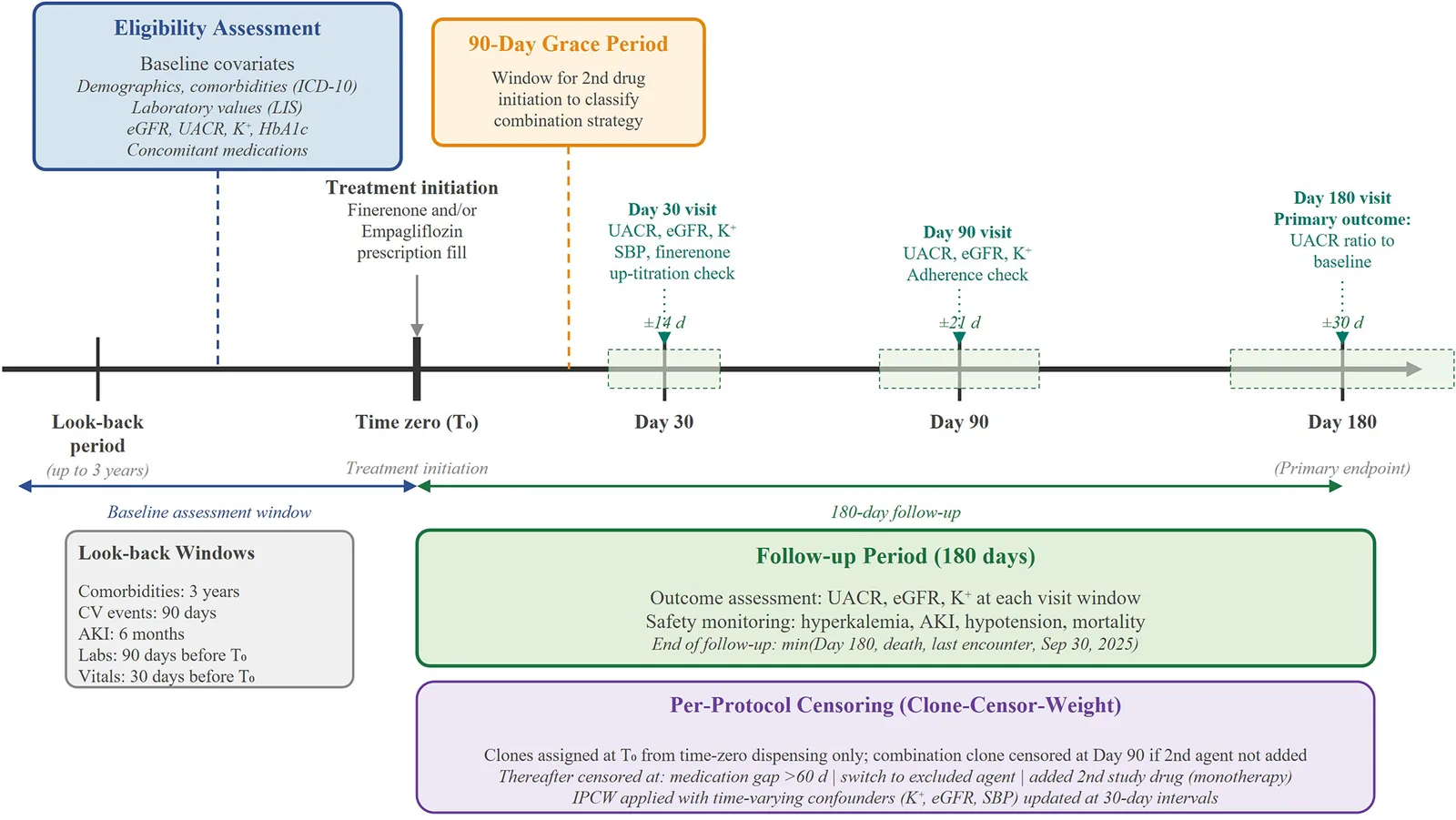
Study timeline and design. Schematic representation of the study design showing the look-back period for baseline covariate assessment, time zero (treatment initiation), the 90-day grace period for combination strategy classification, prespecified visit windows with tolerance ranges (day 30 ± 14 days, day 90 ± 21 days, and day 180 ± 30 days), and the 180-day follow-up period. The per-protocol censoring strategy using the clone-censor-weight approach with inverse probability of censoring weights is shown below the timeline.

### Data source

We used EHR data from three Chinese tertiary hospitals spanning January 2023 to September 2025. Each hospital operates an integrated hospital information system, laboratory information system (LIS), and picture archiving and communication system. Outpatient pharmacy dispensing records, including drug name, dose, quantity, and days’ supply, were used to ascertain treatment exposure. Diagnoses were coded using the International Classification of Diseases, 10th revision (ICD-10). Laboratory values were reported in Chinese standard units (**Supplementary Table S2C**). The complete lists of diagnosis codes, medication codes with Chinese pharmacy names, and laboratory test identifiers are provided in **Supplementary Tables S2A–C**.

### Study population

We identified adults (aged ≥18 years) with concurrent T2D (ICD-10: E11.x) and CKD (ICD-10: N18.x or two estimated glomerular filtration rate [eGFR] values below 60 mL/min/1.73 m² at least 90 days apart) who initiated finerenone, empagliflozin, or both during the study period. The eligibility criteria mirrored the CONFIDENCE trial: eGFR 30–90 mL/min/1.73 m² (calculated using the CKD-EPI 2021 race-free creatinine equation (21)), spot urine UACR 100 to <5,000 mg/g, serum potassium ≤4.8 mmol/L, glycated hemoglobin (HbA1c) <11%, and stable treatment with an ACEi or ARB for ≥1 month. Eligibility was determined exclusively from observed laboratory measurements; no imputed value contributed to the eligibility determination. The qualifying measurement was the value closest to, and on or before, time zero within a 180-day screening window. Analytic baseline covariates were separately defined as the value closest to time zero within a narrower 90-day window, so a patient could satisfy eligibility on an observed qualifying value yet have no measurement inside the narrower analytic window; such values are reported as missing in **Supplementary Table S4** and were multiply imputed for covariate adjustment only. Detailed exclusion criteria are presented in **Supplementary Table S1**. The patient selection process is shown in **Figure 2**.

**Figure 2:**
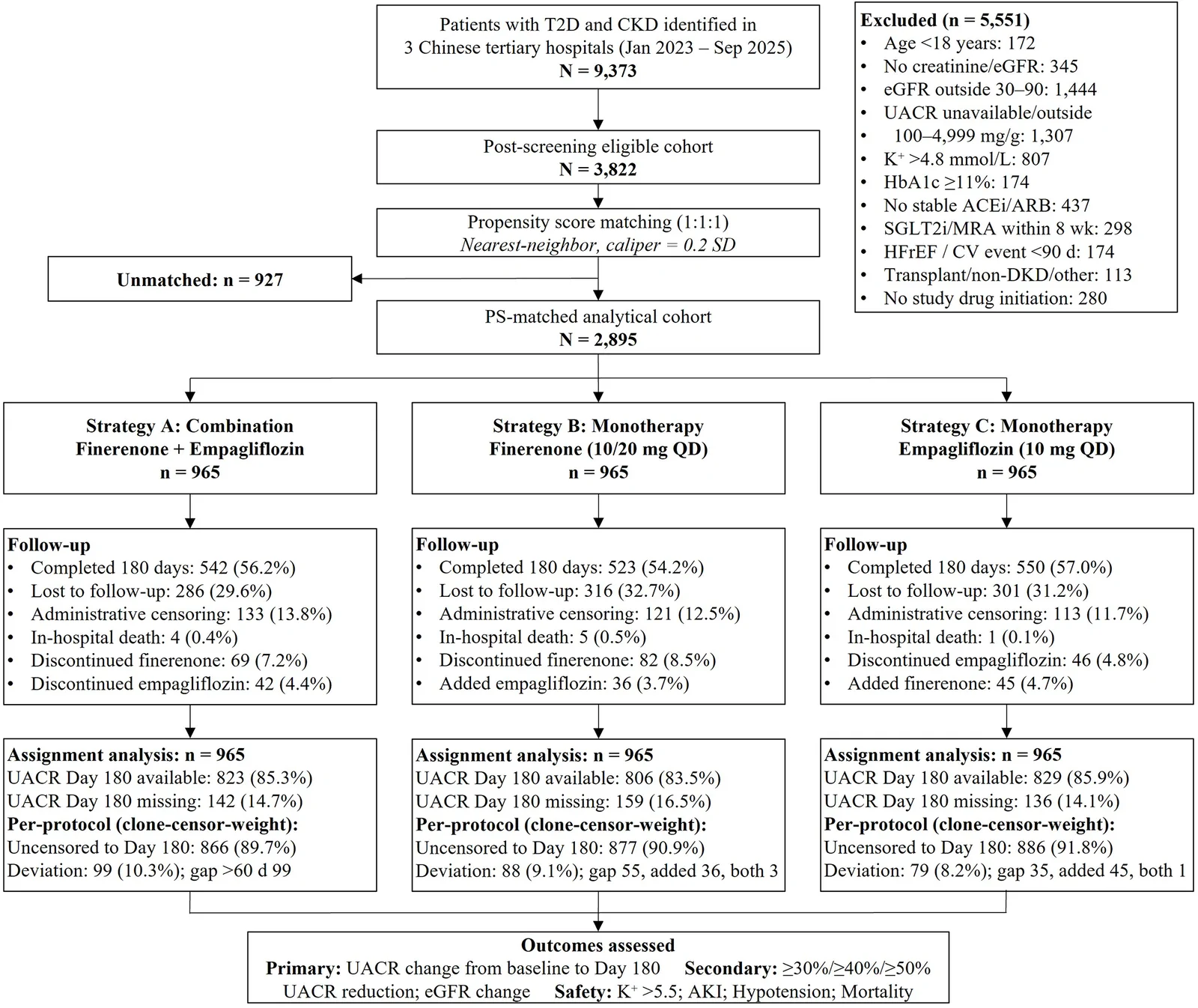
Study flow diagram. Flow of patients from the initial screening pool of 9,373 patients with type 2 diabetes and chronic kidney disease through sequential eligibility assessment, propensity score matching (1:1:1), 180-day follow-up, and analysis populations for both the per-protocol and assignment analyses. Numbers and percentages are shown for each step, including reasons for exclusion, follow-up status, treatment discontinuation, and primary outcome availability by treatment arm.

### Treatment strategies and assignment

Three treatment strategies were compared: (A) finerenone plus empagliflozin initiated within 90 days (combination), (B) finerenone monotherapy, and (C) empagliflozin monotherapy. The combination strategy as emulated permits sequential initiation and is therefore broader than the simultaneous initiation evaluated in CONFIDENCE (9); the present study is accordingly a modified target trial emulation and real-world extension of CONFIDENCE rather than a direct replication. Treatment strategy was determined at time zero (the date of the first qualifying prescription fill) from outpatient pharmacy dispensing records using Chinese drug names (**Supplementary Table S2B**). A 90-day grace period was applied to classify the combination strategy, accommodating sequential initiation common in Chinese clinical practice (20). Finerenone was dosed per the Chinese label: 20 mg once daily if eGFR ≥60, 10 mg if eGFR 25 to <60. A grace period is required whenever the treatment strategies being compared are not initiated instantaneously, and the resulting strategies are dynamic rather than point interventions (22, 23). It carries a specific methodological consequence that determines the estimand hierarchy of this study. Because classification into the combination strategy depends on information accrued after time zero, a patient must survive and remain in care long enough to fill the second prescription; the interval between time zero and second-drug initiation is therefore immortal for that arm (24, 25). An analysis that assigns strategy on this basis is consequently not a true intention-to-treat analysis. As Hernan and Robins have noted, a genuine intention-to-treat contrast is rarely available in observational data, and analyses incorporating a grace period at baseline are directed toward the per-protocol effect of the target trial rather than the effect of assignment (15). We therefore refer to this contrast throughout as the assignment analysis, the observational analogue of an intention-to-treat comparison, and we do not present it as an intention-to-treat effect.

The clone-censor-weight per-protocol analysis is not subject to this bias. Every eligible patient is cloned into all three strategies at time zero, so no clone’s assignment depends on subsequent information and no person-time is immortal by construction; artificial censoring at the point of protocol deviation, with inverse probability of censoring weighting to restore the censored distribution, is the standard remedy (26–28). For this reason, the per-protocol contrast is the estimand on which we base primary inference, and the assignment contrast is reported as a secondary, supportive analysis. Both contrasts were prespecified (**Table 1**). Two further features limit, without eliminating, bias in the assignment contrast: follow-up for all strategies begins at time zero rather than at second-drug initiation, so no person-time is excluded or misclassified into the wrong arm; and grace-period length was varied to 30 and 60 days in the sensitivity analyses (**Supplementary Table S5**). Residual immortal time bias in the assignment contrast cannot be excluded and is addressed in the “Discussion”.

### Outcomes

The primary outcome was the relative change in UACR from baseline to day 180. This was expressed both as a geometric mean ratio (from log-linear regression) and as a percentage change (for descriptive reporting). Two co-primary comparisons were specified: combination versus finerenone and combination versus empagliflozin, with Holm–Bonferroni correction for multiplicity. Secondary outcomes included the proportion achieving ≥30%, ≥40%, and ≥50% UACR reduction at day 180 and absolute change in eGFR at days 30 and 180. Safety outcomes included hyperkalemia (serum potassium >5.5 mmol/L), severe hyperkalemia (>6.0 mmol/L), acute kidney injury (AKI), eGFR decline >30% at day 30, symptomatic hypotension, genital mycotic infection, diabetic ketoacidosis, severe hypoglycemia, heart failure (HF) hospitalization, and in-hospital mortality (**Table 2**). The outcome definitions are detailed in the **Supplementary Materials**.

**Table 2:** Safety outcomes in the PS-matched cohort.

Safety outcome	Combination (*n* = 965)	Finerenone (*n* = 965)	Empagliflozin (*n* = 965)
Hyperkalemia (K^+^ >5.5 mmol/L), *n* (%)	123 (12.7)	124 (12.8)	34 (3.5)
Severe hyperkalemia (K^+^ >6.0 mmol/L), *n* (%)	12 (1.2)	10 (1.0)	1 (0.1)
Hyperkalemia requiring intervention, *n* (%)	44 (4.6)	39 (4.0)	16 (1.7)
eGFR decline >30% at day 30, *n* (%)	8 (0.8)	1 (0.1)	7 (0.7)
Acute kidney injury, *n* (%)	47 (4.9)	27 (2.8)	22 (2.3)
Symptomatic hypotension, *n* (%)	18 (1.9)	6 (0.6)	4 (0.4)
Genital mycotic infection, *n* (%)	15 (1.6)	0 (0.0)	14 (1.5)
Diabetic ketoacidosis, *n* (%)	2 (0.2)	3 (0.3)	1 (0.1)
Severe hypoglycemia, *n* (%)	21 (2.2)	12 (1.2)	14 (1.5)
In-hospital mortality, *n* (%)	4 (0.4)	5 (0.5)	1 (0.1)
Heart failure hospitalization, *n* (%)	13 (1.3)	17 (1.8)	20 (2.1)
Any serious adverse event, *n* (%)	83 (8.6)	78 (8.1)	72 (7.5)
Potassium levels during follow-up			
Maximum K^+^ during FU, mmol/L, mean ± SD	5.0 ± 0.5	5.0 ± 0.5	4.7 ± 0.4

Diagnosis-code-defined outcomes (acute kidney injury, symptomatic hypotension, genital mycotic infection, diabetic ketoacidosis, severe hypoglycemia, heart failure hospitalization, in-hospital mortality, and any serious adverse event) were ascertained for all 965 patients per arm from complete inpatient and outpatient diagnosis records. Laboratory-defined outcomes (hyperkalemia, severe hyperkalemia, eGFR decline >30% at day 30, and maximum potassium during follow-up) were ascertained among patients with at least one corresponding follow-up measurement; availability of follow-up potassium and eGFR values by arm is reported in **Supplementary Table S4**. A serious adverse event was defined per ICH E2A as any event that resulted in death, was life-threatening, required inpatient hospitalization or prolongation of existing hospitalization, resulted in persistent or significant disability, or was judged an important medical event; these were identified from inpatient admission records and associated ICD-10 discharge diagnoses. Hyperkalemia requiring intervention was defined as serum potassium >5.5 mmol/L accompanied by at least one of the following: dose reduction or discontinuation of finerenone or another renin–angiotensin–aldosterone system inhibitor within 14 days, dispensing of a potassium binder (**Supplementary Table S2B**), or an emergency department visit or hospitalization with a hyperkalemia diagnosis code. Categories are not mutually exclusive; a patient contributing more than one qualifying event is counted once.

eGFR, estimated glomerular filtration rate; FU, follow-up; K^+^, serum potassium; SAE, serious adverse event.

### Follow-up

Follow-up began at time zero and continued until the earliest of 180 days, in-hospital death, last clinical encounter (a gap exceeding 90 days defined loss to follow-up), or administrative end of study (September 30, 2025). Three distinct quantities are reported separately and should not be conflated: completion of the full 180 days of observation, loss to follow-up or administrative censoring before day 180, and availability of a day-180 UACR measurement. Because the day-180 visit window extends to plus or minus 30 days, a measurement obtained from day 150 onward qualifies as a day-180 value even when observation ended before day 180; consequently, the proportion with an available day-180 UACR exceeds the proportion completing the full 180 days (**Supplementary Table S4**). Laboratory values were captured within prespecified visit windows: day 30 (± 14 days), day 90 (± 21 days), and day 180 (± 30 days). Because laboratory-based outcomes can only be detected when the corresponding test is performed, outcome-specific denominators differ between laboratory-defined and diagnosis-code-defined outcomes; these denominators are reported in **Table 2** and the underlying availability of follow-up measurements in **Supplementary Table S4**. Serum creatinine and urine albumin were measured on each hospital’s own accredited clinical platform; all three laboratories participate in the Chinese National Center for Clinical Laboratories external quality assessment scheme, and creatinine assays were isotope-dilution mass spectrometry-traceable. Because assay calibration was not additionally harmonized centrally for this study, residual between-hospital measurement variation cannot be excluded.

### Statistical analysis

Two estimands were prespecified (**Table 1**). The primary estimand was the per-protocol effect, that is, the effect of initiating and sustaining the assigned strategy, estimated by the clone–censor–weight approach described below. The secondary estimand was the effect of strategy assignment among the matched population, the observational analogue of an intention-to-treat effect. Where an analysis is described below as the assignment analysis, this secondary estimand is meant. For the assignment analysis, we used 1:1:1 propensity score (PS) matching across the three treatment strategies (29). A multinomial logistic regression model was fitted to estimate the generalized PS, incorporating all baseline covariates listed in **Table 3**. Nearest-neighbor matching with a caliper of 0.2 standard deviations of the logit PS was performed. Covariate balance was assessed using standardized mean differences (SMDs), with a threshold of <0.10 indicating adequate balance (**Supplementary Table S3**; **Supplementary Figure S2**) (29, 30). Covariates retaining an SMD value ≥0.10 after matching are reported explicitly and considered as potential sources of residual confounding (31). Positivity was assessed by examining the overlap of PS distributions across treatment arms (**Supplementary Materials S4**). The primary outcome was analyzed using linear regression on the log-transformed UACR ratio within the PS-matched cohort, with robust standard errors, yielding least-squares mean ratios with 95% confidence intervals (CIs). Categorical secondary outcomes were compared using risk differences. Missing baseline covariates were handled using multiple imputations by chained equations with 20 imputed datasets under the missing-at-random assumption (**Supplementary Table S4**) (32). For the primary outcome, the analytic dataset supplied a single derived day-180 UACR value for the 437 matched patients (15.1%) without an observed measurement in the day-180 window; these values were generated at dataset construction, and the tipping-point analysis (**Supplementary Table S9**) directly evaluates sensitivity to their assumed values.

**Table 3:** Baseline characteristics of the propensity-score-matched cohort.

Characteristic	Combination (*n* = 965)	Finerenone (*n* = 965)	Empagliflozin (*n* = 965)	SMD
Demographics				
Age, years, mean ± SD	59.6 ± 9.6	59.8 ± 10.2	59.4 ± 9.9	0.043
Male sex, *n* (%)	600 (62.2)	588 (60.9)	606 (62.8)	0.038
BMI, kg/m², mean ± SD	25.5 ± 5.1	25.4 ± 5.3	25.2 ± 5.2	0.051
Body weight, kg, mean ± SD	68.4 ± 12.3	68.2 ± 12.6	68.1 ± 12.3	0.024
Current smoker, *n* (%)	202 (26.7)	205 (27.6)	197 (26.3)	0.025
Insurance type				
UEBMI	537 (56.2)	540 (56.4)	550 (57.3)	0.037
URBMI	200 (20.9)	172 (18.0)	187 (19.5)	0.037
NRCMS	180 (18.8)	199 (20.8)	163 (17.0)	0.037
Self-pay/other	39 (4.1)	47 (4.9)	60 (6.2)	0.037
Kidney function				
eGFR, mL/min/1.73 m², mean ± SD	46.8 ± 13.2	45.7 ± 12.6	46.6 ± 12.8	0.085
eGFR <45, *n* (%)	504 (52.2)	523 (54.2)	499 (51.7)	0.085
eGFR 45–<60, *n* (%)	307 (31.8)	313 (32.4)	330 (34.2)	0.085
eGFR ≥60, *n* (%)	137 (14.2)	120 (12.4)	127 (13.2)	0.085
eGFR missing, *n* (%)	17 (1.8)	9 (0.9)	9 (0.9)	—
UACR, mg/g, median (IQR)	585.2 (316.8–1,171.7)	569.3 (300.4–1,108.0)	587.0 (320.7–1,096.5)	0.053
Serum creatinine, μmol/L, mean ± SD	142.3 ± 33.5	144.5 ± 33.0	142.8 ± 32.0	0.066
Metabolic parameters				
HbA1c, %, mean ± SD	7.7 ± 1.3	7.7 ± 1.3	7.7 ± 1.3	0.033
Fasting glucose, mmol/L, mean ± SD	7.9 ± 2.5	7.8 ± 2.3	7.9 ± 2.5	0.063
Serum potassium, mmol/L, mean ± SD	4.3 ± 0.3	4.3 ± 0.3	4.3 ± 0.3	0.060
Blood pressure and hematology				
SBP, mmHg, mean ± SD	137.8 ± 15.7	137.9 ± 16.0	137.6 ± 16.0	0.018
DBP, mmHg, mean ± SD	80.0 ± 10.0	79.7 ± 9.8	80.2 ± 9.9	0.050
Hemoglobin, g/L, mean ± SD	119.0 ± 16.8	118.2 ± 16.4	118.6 ± 16.5	0.048
Serum albumin, g/L, mean ± SD	28.8 ± 3.8	29.0 ± 3.9	28.7 ± 3.9	0.069
Total cholesterol, mmol/L, mean ± SD	4.8 ± 1.1	4.9 ± 1.1	4.8 ± 1.1	0.085
LDL cholesterol, mmol/L, mean ± SD	2.8 ± 0.9	2.6 ± 0.9	2.7 ± 0.9	0.117
Triglycerides, mmol/L, median (IQR)	1.6 (1.2–2.1)	1.6 (1.2–2.1)	1.6 (1.2–2.2)	0.074
Comorbidities				
Hypertension, *n* (%)	900 (93.3)	889 (92.1)	910 (94.3)	0.087
ASCVD, *n* (%)	561 (58.1)	578 (59.9)	563 (58.3)	0.036
Coronary artery disease, *n* (%)	289 (29.9)	281 (29.1)	301 (31.2)	0.045
Myocardial infarction, *n* (%)	52 (5.4)	54 (5.6)	59 (6.1)	0.031
Stroke, *n* (%)	212 (22.0)	242 (25.1)	214 (22.2)	0.073
Heart failure, *n* (%)	58 (6.0)	51 (5.3)	54 (5.6)	0.031
Atrial fibrillation, *n* (%)	45 (4.7)	43 (4.5)	47 (4.9)	0.020
Diabetic retinopathy, *n* (%)	461 (47.8)	460 (47.7)	462 (47.9)	0.004
COPD, *n* (%)	70 (7.3)	76 (7.9)	84 (8.7)	0.054
T2D duration, years, mean ± SD	13.1 ± 6.7	13.1 ± 6.7	12.7 ± 6.8	0.058
Concomitant medications				
ACE inhibitor, *n* (%)	102 (10.6)	119 (12.3)	105 (10.9)	0.055
ARB, *n* (%)	863 (89.4)	846 (87.7)	860 (89.1)	0.055
Metformin, *n* (%)	357 (37.0)	347 (36.0)	344 (35.6)	0.028
Insulin, *n* (%)	727 (75.3)	737 (76.4)	720 (74.6)	0.041
DPP-4 inhibitor, *n* (%)	117 (12.1)	86 (8.9)	124 (12.8)	0.127
GLP-1 receptor agonist, *n* (%)	79 (8.2)	84 (8.7)	86 (8.9)	0.026
Sulfonylurea, *n* (%)	150 (15.5)	168 (17.4)	149 (15.4)	0.053
Calcium channel blocker, *n* (%)	457 (47.4)	472 (48.9)	486 (50.4)	0.060
Beta-blocker, *n* (%)	239 (24.8)	243 (25.2)	227 (23.5)	0.039
Diuretic, *n* (%)	159 (16.5)	164 (17.0)	160 (16.6)	0.014
Statin, *n* (%)	516 (53.5)	492 (51.0)	496 (51.4)	0.050
Antiplatelet agent, *n* (%)	281 (29.1)	289 (29.9)	289 (29.9)	0.018

ACEi, angiotensin-converting enzyme inhibitor; ARB, angiotensin receptor blocker; ASCVD, atherosclerotic cardiovascular disease; BMI, body mass index; COPD, chronic obstructive pulmonary disease; DBP, diastolic blood pressure; DPP-4, dipeptidyl peptidase-4; eGFR, estimated glomerular filtration rate; GLP-1, glucagon-like peptide-1; HbA1c, glycated hemoglobin; IQR, interquartile range; LDL, low-density lipoprotein; NRCMS, New Rural Cooperative Medical Scheme; SBP, systolic blood pressure; SD, standard deviation; SMD, standardized mean difference; T2D, type 2 diabetes; UACR, urine albumin-to-creatinine ratio; UEBMI, Urban Employee Basic Medical Insurance; URBMI, Urban Resident Basic Medical Insurance.

For the primary per-protocol effect, we applied the clone-censor-weight approach (28, 33). Each eligible patient was cloned into all three treatment arms at time zero. A clone was censored only when the observed treatment history first became inconsistent with its assigned strategy and never on the basis of information accrued after time zero. At time zero, the only information used was which drug or drugs were dispensed on that date. A patient who initiated a single agent, therefore, remained compatible with and uncensored under both the corresponding monotherapy strategy and the combination strategy because the second agent could still be added within the grace period; only the clone assigned to the monotherapy strategy of the agent not dispensed at time zero was censored at time zero. A patient who initiated both agents on the same date remained uncensored under the combination strategy, and both monotherapy clones were censored at time zero. Thereafter, the combination clone was censored at day 90 if the second agent had not been dispensed by then, and each clone was censored at the first subsequent deviation from its assigned strategy, defined as a medication gap exceeding 60 days in any assigned agent, switching to an excluded agent, or, for a monotherapy strategy, addition of the second study drug. Inverse probability of censoring weights (IPCW) with time-varying confounders (serum potassium, eGFR, and systolic blood pressure [SBP] updated at 30-day intervals) were applied to adjust for informative censoring. Weights were stabilized and truncated at the 1st and 99th percentiles. CIs were estimated using 500 bootstrap iterations (**Supplementary Table S8**). Inverse probability of censoring weighting recovers an unbiased per-protocol estimate only if censoring is independent of the outcome conditional on the measured baseline and time-varying covariates included in the weight model (34); this assumption is not testable and is considered in the “Discussion”.

### Sensitivity and subgroup analyses

We conducted 10 prespecified sensitivity analyses (**Supplementary Table S5**; **Supplementary Figure S4**): restricting to UACR ≥300 mg/g (the original CONFIDENCE threshold), varying the grace period to 30 and 60 days, complete case analysis, inverse probability of treatment weighting (IPTW, non-stabilized, and stabilized and trimmed) as an alternative to matching, as-treated analysis, per-protocol analysis with IPCW, a landmark analysis excluding the first 14 days, and a per-protocol analysis restricted to simultaneous initiation of both agents. We computed E-values to assess robustness to unmeasured confounding (35). Prespecified subgroup analyses were performed by baseline eGFR (<60 versus ≥60), UACR (≤850 versus >850 mg/g), age (<65 versus ≥65), sex, HbA1c (<7.5% versus ≥7.5%), and atherosclerotic cardiovascular disease (ASCVD) status, with *P*-values for interaction (**Supplementary Table S6**; **Supplementary Figure S3**).

### Ethics approval

The study protocol was reviewed and approved by the Ethics Committee of Deyang Hospital affiliated to Chengdu University of Traditional Chinese Medicine (approval number 2025-06-006-KDD), which acted as the reviewing committee of record. The two other participating hospitals formally accepted this approval under a reliance arrangement and did not issue separate approval numbers. Informed consent was waived by the reviewing committee because the study used retrospective, de-identified EHR data with no direct patient contact.

### Patient and public involvement

Patients or members of the public were not involved in the design, conduct, reporting, or dissemination plans of this study. The research questions and outcome measures were developed based on the protocol of the emulated trial (CONFIDENCE) and established clinical endpoints in DKD.

## Results

### Study population

Of 9,373 patients with T2D and CKD identified across the three hospitals, 5,551 were excluded during eligibility screening (**Figure 2**; **Supplementary Table S1**). The most common reasons for exclusion were eGFR outside the 30–90 mL/min/1.73 m² range (*n* = 1,444, 16.3% of those assessed at that step), UACR outside the eligible range (*n* = 1,307, 17.6%), and serum potassium >4.8 mmol/L (*n* = 807, 13.2%). After applying all criteria, 3,822 patients were eligible and had initiated at least one study drug. PS matching yielded 2,895 patients (965 per arm) for the primary analysis; 927 remained unmatched.

### Baseline characteristics

**Table 3** presents the baseline characteristics of the matched cohort. The participants had a mean age of 59.6 years (standard deviation [SD] 9.9), 62.0% were men, and mean BMI was 25.4 kg/m². Kidney function was moderately impaired: mean eGFR 46.4 mL/min/1.73 m² (SD 12.9) and median UACR 583 mg/g (interquartile range [IQR] 313–1,124). Mean serum potassium was 4.30 mmol/L (SD 0.32), mean HbA1c was 7.7% (SD 1.3), and mean hemoglobin was 118.6 g/L (SD 16.6). Hypertension was present in 93.2%, ASCVD in 58.8%, and diabetic retinopathy in 47.8%. ARBs were used by 88.7% and insulin by 75.4%, reflecting typical Chinese prescribing patterns (13). After matching, 25 of the 27 propensity score model covariates achieved a maximum pairwise SMD below 0.10. Two covariates retained residual imbalance: dipeptidyl peptidase-4 (DPP-4) inhibitor use (SMD 0.127) and, among variables not included in the propensity score model, low-density lipoprotein (LDL) cholesterol (SMD 0.117) (**Table 3**; **Supplementary Table S3**; **Supplementary Figure S2**). Baseline eGFR was missing for 17, nine, and nine patients in the combination, finerenone, and empagliflozin arms, respectively; the eGFR category counts in **Table 3** are, therefore, reported among patients with a recorded baseline value. The overall missing data rate was 15.1% (**Supplementary Table S4**).

### Primary outcome

At day 180, the median UACR reduction from baseline was 52.0% in the combination arm, 32.1% in the finerenone arm, and 28.9% in the empagliflozin arm (**Table 4**; **Figure 3**). In the adjusted analysis, the mean difference in UACR percent change between the combination and finerenone arms was −19.6 percentage points (95% CI −20.5 to −18.7; *P* < 0.001) and between the combination and empagliflozin arms was −22.9 percentage points (95% CI −23.8 to −22.0; *P* < 0.001); both remained significant after Holm–Bonferroni correction. The least-squares geometric mean UACR ratio to baseline was 0.48 for the combination, 0.68 for finerenone, and 0.71 for empagliflozin (**Table 4**). UACR trajectories showed progressive separation from day 30 onwards (**Figure 3**).

**Table 4:** Primary and secondary efficacy outcomes in the PS-matched cohort (assignment analysis).

Outcome	Combination (*n* = 965)	Finerenone (*n* = 965)	Empagliflozin (*n* = 965)	Combo vs. finerenone difference (95% CI)	Combo vs. empagliflozin difference (95% CI)
Primary outcome					
UACR change at day 180, %, mean ± SD	-51.5 ± 10.2	-32.0 ± 10.0	-28.6 ± 10.2	-19.6 (-20.5 to -18.7) *P* < 0.001	-22.9 (-23.8 to -22.0) *P* < 0.001
UACR change at day 180, %, median (IQR)	-52.0 (-58.8–44.6)	-32.0 (-38.7–25.7)	-28.8 (-35.8–21.5)	—	—
UACR LS mean ratio to baseline	0.48	0.68	0.71	Ratio: 0.71	Ratio: 0.68
Secondary outcomes, UACR response					
UACR reduction ≥30%, *n* (%)	943 (97.7)	564 (58.4)	440 (45.6)	RD: +39.3 pp	RD: +52.1 pp
UACR reduction ≥40%, *n* (%)	852 (88.3)	198 (20.5)	138 (14.3)	RD: +67.8 pp	RD: +74.0 pp
UACR reduction ≥50%, *n* (%)	539 (55.9)	31 (3.2)	10 (1.0)	RD: +52.6 pp	RD: +54.8 pp
Secondary outcomes, eGFR change					
eGFR change at day 30, mL/min/1.73 m²	-4.6 ± 3.0	-2.4 ± 3.0	-3.0 ± 3.0	-2.2 (-2.4 to -1.9)	-1.6 (-1.8 to -1.3)
eGFR change at day 180, mL/min/1.73 m²	-1.5 ± 3.0	-0.5 ± 3.0	-1.1 ± 2.9	-1.0 (-1.3 to -0.7)	-0.4 (-0.7 to -0.1)

CI, confidence interval; eGFR, estimated glomerular filtration rate; IQR, interquartile range; LS, least squares; pp, percentage points; PS, propensity score; RD, risk difference; SD, standard deviation; UACR, urine albumin-to-creatinine ratio.

**Figure 3:**
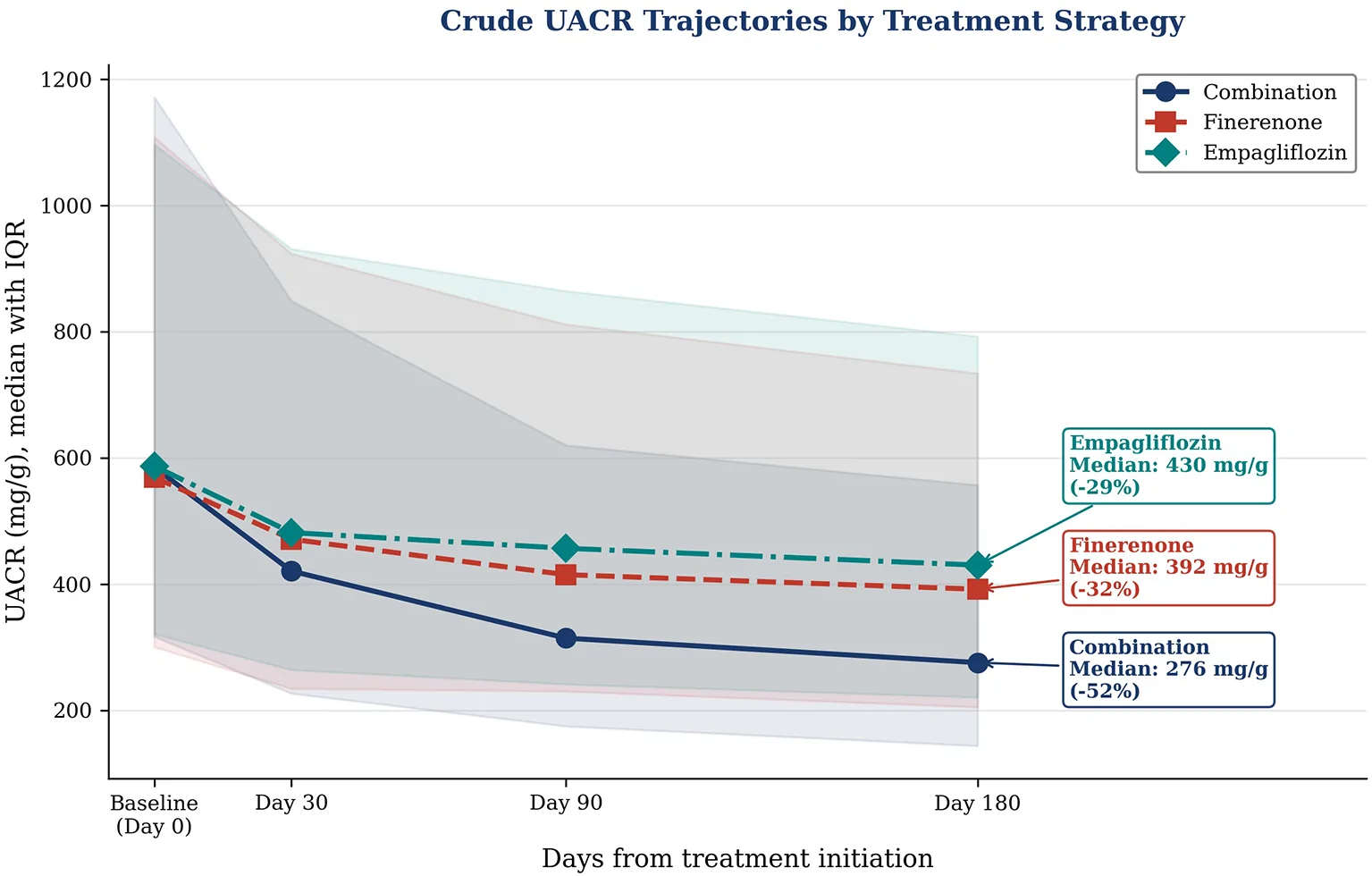
Crude urine albumin-to-creatinine ratio trajectories by treatment strategy. Median values with interquartile range shading at baseline, day 30, day 90, and day 180 for the combination (finerenone plus empagliflozin), finerenone monotherapy, and empagliflozin monotherapy arms. Annotated endpoint values show the median and percentage change from baseline at day 180.

In the per-protocol clone–censor–weight analysis, which constitutes the primary causal contrast because it is not susceptible to immortal time bias arising from the grace period, the difference in UACR change at day 180 was −19.5 percentage points (95% CI −20.5 to −18.5) for combination versus finerenone and −22.8 percentage points (95% CI −23.8 to −21.8) for combination versus empagliflozin (**Supplementary Table S8**). Under the corrected censoring rule, 866 combination clones (89.7%), 877 finerenone clones (90.9%), and 886 empagliflozin clones (91.8%) remained uncensored to day 180; deviations comprised medication gaps in all three strategies (99, 55, and 35 patients) and addition of the second study drug in the monotherapy strategies (36 and 45 patients), with three patients and one patient meeting both criteria (**Supplementary Table S8**). The per-protocol and assignment estimates were closely concordant, which is consistent with, though it does not by itself demonstrate, a limited contribution of grace-period immortal time to the assignment contrast. Among combination-strategy patients, the second agent was added a median of 14 days after time zero (interquartile range 0 to 30; range 0 to 60); 325 patients (33.7%) initiated both agents on the same day, 92 (9.5%) within 1 to 7 days, 338 (35.0%) within 8 to 30 days, and 210 (21.8%) within 31 to 60 days. No patient initiated the second agent between 61 and 90 days, so the grace period was never exercised beyond 60 days. In a prespecified sensitivity analysis restricted to simultaneous initiation, the per-protocol differences were −20.4 percentage points for combination versus finerenone and −23.8 percentage points for combination versus empagliflozin, consistent with the primary analysis (**Supplementary Table S5**).

### Secondary outcomes

The proportion of patients achieving a UACR reduction of ≥30% at day 180 was substantially higher in the combination arm than in either monotherapy arm (**Table 4**; **Figure 4**). eGFR declined initially in all three arms at day 30, consistent with the known hemodynamic dip, with the largest decline in the combination arm (**Supplementary Figure S5**). Because the combination strategy permitted second-drug initiation within a 90-day grace period, a proportion of combination-arm patients had not yet started the second agent by day 30; day-30 outcomes in this arm therefore reflect a mixture of single-agent and dual-agent exposure and should not be interpreted as effects of established combination therapy. Sensitivity analyses restricting the grace period to 30 days yielded consistent day-180 estimates (**Supplementary Table S5**). By day 180, eGFR had partially recovered, with a mean difference between the combination and finerenone arms of −1.0 mL/min/1.73 m² (95% CI −1.3 to −0.7) (**Table 4**; **Figure 4**).

**Figure 4:**
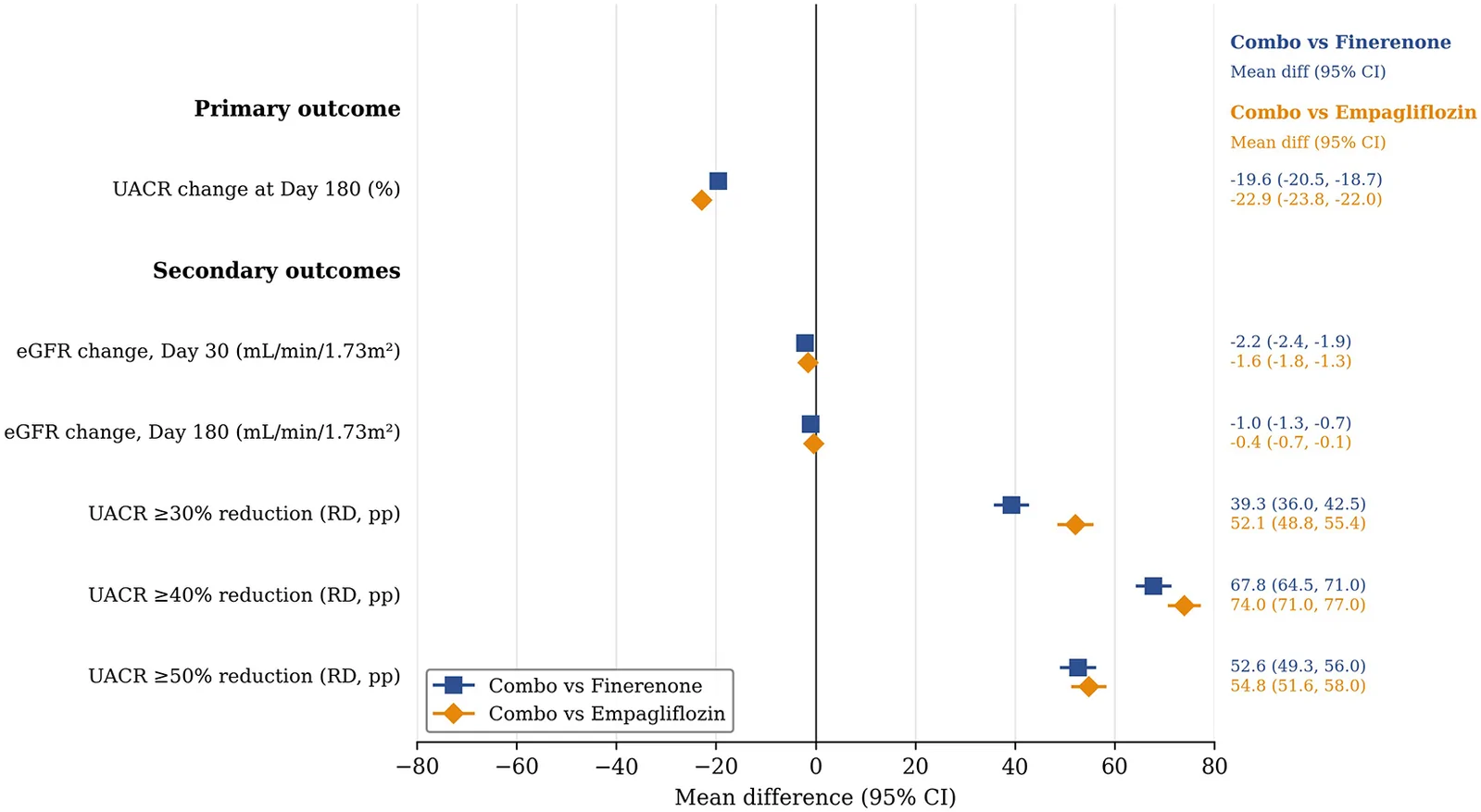
Forest plot of primary and secondary efficacy outcomes (assignment analysis). Mean differences with 95% confidence intervals for the combination versus finerenone (blue) and combination versus empagliflozin (orange) comparisons. Outcomes include urine albumin-to-creatinine ratio change at day 180 (primary), estimated glomerular filtration rate change at days 30 and 180, and proportions achieving ≥30%, ≥40%, and ≥50% urine albumin-to-creatinine ratio reduction (risk differences in percentage points).

### Safety outcomes

Hyperkalemia (serum potassium >5.5 mmol/L) occurred in 12.7% of the combination arm, 12.8% of the finerenone arm, and 3.5% of the empagliflozin arm (**Table 2**; **Figure 5**). Severe hyperkalemia (>6.0 mmol/L) was infrequent across all arms (0.1%–1.2%). Serum potassium rose in finerenone-containing arms from day 30 onwards but stabilized thereafter (**Supplementary Figure S6**). AKI occurred in 4.9%, 2.8%, and 2.3% of the combination, finerenone, and empagliflozin arms, respectively. In-hospital mortality was low (0.1%–0.5%) (**Table 2**; **Figure 5**). Diagnosis-code-defined safety outcomes were ascertained for all 965 patients per arm, whereas laboratory-defined outcomes were conditional on the availability of follow-up measurements (**Table 2** footnote; **Supplementary Table S4**).

**Figure 5:**
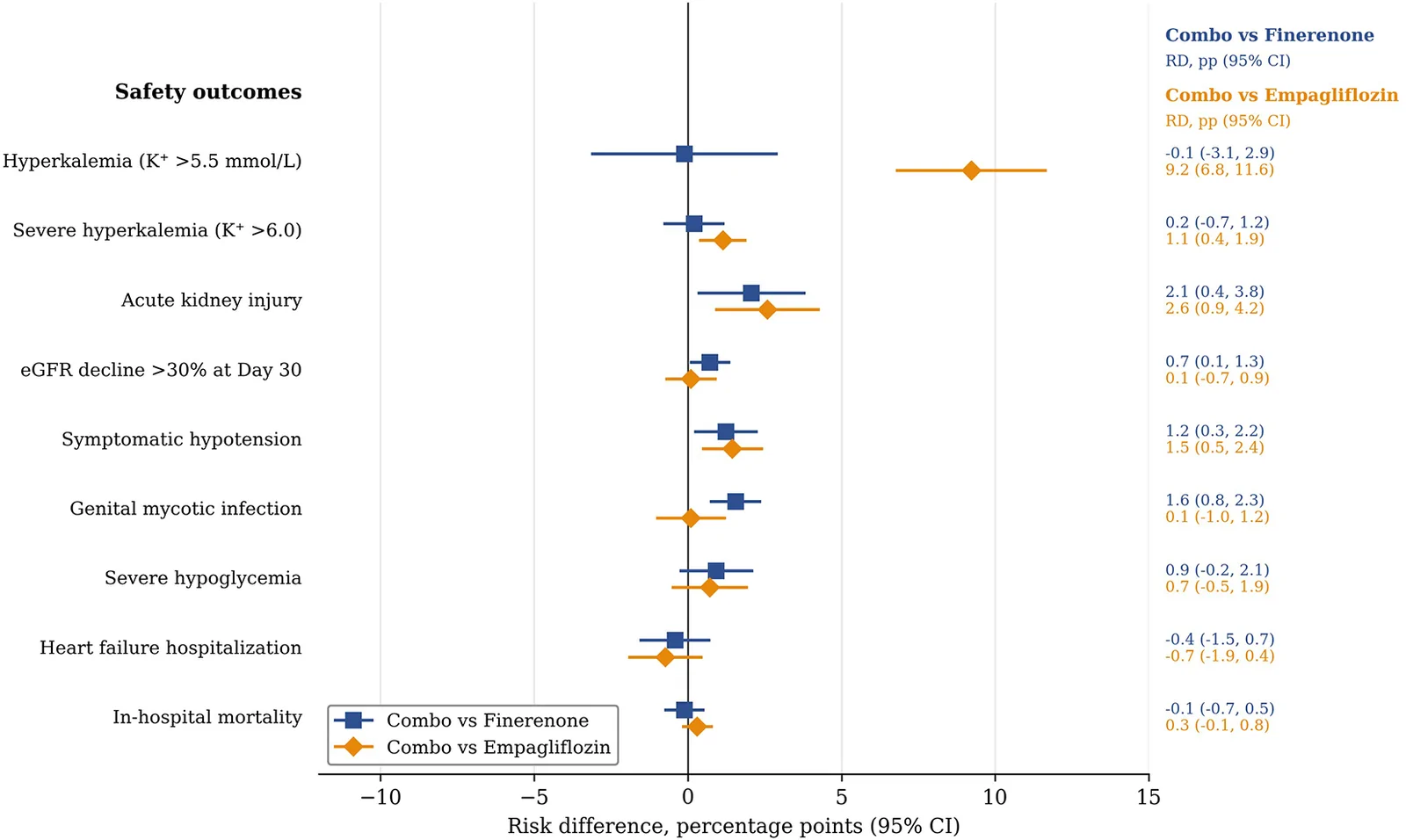
Forest plot of safety outcomes by treatment strategy. Risk differences in percentage points with 95% confidence intervals for the combination versus finerenone (blue) and combination versus empagliflozin (orange) comparisons. Safety outcomes include hyperkalemia, severe hyperkalemia, acute kidney injury, estimated glomerular filtration rate decline exceeding 30% at day 30, symptomatic hypotension, genital mycotic infection, severe hypoglycemia, heart failure hospitalization, and in-hospital mortality.

### Sensitivity, subgroup, and per-protocol analyses

Observation for the full 180 days was completed by 542 (56.2%), 523 (54.2%), and 550 (57.0%) patients in the combination, finerenone, and empagliflozin strategies; loss to follow-up accounted for 286, 316, and 301 patients, administrative censoring for 133, 121, and 113, and death for four, five, and one. A day-180 UACR value was nevertheless available for 823 (85.3%), 806 (83.5%), and 829 (85.9%) patients because 1,102 patients whose observation ended before day 180 (median 160 days) had a qualifying measurement within the plus or minus 30-day visit window (**Supplementary Table S4**). The results were consistent across all 10 sensitivity analyses (**Supplementary Table S5**; **Supplementary Figure S4**). The subgroup analyses demonstrated consistent treatment effects with no significant interactions (all *P*-interaction >0.30) (**Supplementary Table S6**; **Supplementary Figure S3**). The primary per-protocol estimates are reported above. Both per-protocol contrasts are reported in **Supplementary Table S8**, and the per-protocol row of **Supplementary Table S5** has been corrected to the same values so that the two tables agree. Emulated treatment effects were broadly consistent with the CONFIDENCE trial, although the study population was younger (mean, 59.6 versus 68 years) and had lower baseline eGFR (46.4 versus 54.2 mL/min/1.73 m²) (**Supplementary Table S7**).

## Discussion

In this emulation of the CONFIDENCE trial using EHR data from three Chinese tertiary hospitals, the combination of finerenone and empagliflozin reduced UACR by 52% at day 180. In the per-protocol clone–censor–weight analysis, on which our primary inference rests because it is not susceptible to immortal time bias from the grace period, combination therapy lowered UACR by 19.5 percentage points more than finerenone and 22.8 percentage points more than empagliflozin. This magnitude closely matched the original trial (9). Finerenone alone reduced UACR by 32% and empagliflozin alone by 29%, again consistent with the trial. These results suggest that the additive albuminuria-lowering effect of nsMRA-SGLT2 inhibitor combination therapy is reproducible in Chinese clinical practice.

The choice of estimand deserves emphasis because it determines how much weight each of our estimates can bear. The grace period that permits sequential initiation and which reflects how combination therapy is actually started in Chinese practice means that assignment to the combination strategy uses information that accrues after time zero. The resulting assignment contrast is therefore not an intention-to-treat effect in the sense understood in randomized trials and should not be interpreted as one (15). It is also worth noting that an intention-to-treat analysis is not automatically the conservative choice: under incomplete adherence, it may be biased toward or away from the null depending on the pattern of non-adherence, so its apparent conservatism cannot be assumed (36). We have accordingly based our primary inference on the per-protocol effect estimated by cloning, censoring, and weighting, which eliminates grace-period immortal time by construction (26, 28), and we present the assignment contrast as supportive. The two estimates were closely concordant, but concordance is reassuring rather than probative: it is consistent with limited immortal time bias, yet it does not exclude it, and the per-protocol estimate itself remains valid only under the assumption that censoring is independent of outcome given the measured covariates in the weight model (34).

The concordance between our emulated estimates and the CONFIDENCE trial strengthens confidence in both the TTE methodology and the generalizability of the trial findings. Our population differed from CONFIDENCE in several respects: patients were nearly a decade younger (mean, 59.6 versus 68 years), had lower BMI (25.4 versus approximately 31 kg/m²), and overwhelmingly used ARBs rather than ACEi (88% versus approximately 30% in global trials) (12, 37). Despite these differences, the treatment effects were remarkably similar. This directionally aligns with the FIDELIO-DKD China subgroup, in which the kidney composite hazard ratio was 0.59 (95% CI 0.39–0.88); however, this estimate derives from a small prespecified subgroup (*n* = 372) with a wide confidence interval that overlaps the overall FIDELIO-DKD estimate (hazard ratio 0.82, 95% CI 0.73–0.93), so the apparently larger effect in Chinese patients should be interpreted cautiously and may reflect subgroup variability rather than a true ethnicity-specific difference (12).

The safety profile requires careful interpretation rather than reassurance. The hyperkalemia rates in finerenone-containing arms (12.7–12.8%) were lower than in the CONFIDENCE trial (15.3–18.6%) (9), possibly reflecting tighter baseline potassium eligibility in routine clinical practice. However, acute kidney injury (4.9% versus 2.8% and 2.3%) and symptomatic hypotension (1.9% versus 0.6% and 0.4%) were numerically more frequent with combination therapy, and these differences are clinically relevant even though the study was not powered for formal safety comparisons. Importantly, adding empagliflozin to finerenone did not meaningfully attenuate hyperkalemia risk compared with finerenone alone. This is consistent with the CONFIDENCE secondary hyperkalemia analysis and underscores that potassium monitoring remains essential regardless of SGLT2 inhibitor coadministration (9). The initial eGFR dip in the combination arm was reversible by day 90 (**Supplementary Figure S5**).

### Implications for clinical practice in China

The 2023 Chinese expert consensus on finerenone endorsed its use in DKD patients on background RAS inhibition but provided limited guidance on the combination with SGLT2 inhibitors (38). Our findings provide the first real-world data supporting this combination in Chinese patients. ARB-based RAS inhibition predominates in our cohort (88.7%) because of the substantially higher incidence of ACEi-associated cough in patients of Chinese descent, in whom discontinuation for cough is approximately 2.5-fold more common than in general populations (39). This difference in background RAS blockade is relevant to cross-study comparison of hyperkalemia: ACEi and ARB differ in their effect on aldosterone escape and in observed hyperkalemia reporting, with pharmacovigilance data suggesting a relatively greater hyperkalemia signal for ACEi than for ARB (40). An ARB-predominant cohort may therefore have a modestly lower baseline hyperkalemia propensity than the ACEi-containing CONFIDENCE population, and this, together with the lower baseline potassium permitted at entry and possible differences in dietary potassium intake, may contribute to the lower rates that we observed. These mechanisms are hypotheses generated by the present comparison rather than tested findings. Whatever the explanation, an absolute hyperkalemia rate of 12.7% with combination therapy versus 3.5% with empagliflozin alone is not trivial in routine practice. Our data support structured potassium surveillance when finerenone is added to an SGLT2 inhibitor in Chinese tertiary care, with serum potassium and creatinine measured before initiation, within 4 weeks of initiation or dose escalation and periodically thereafter, consistent with KDIGO 2024 guidance (41). Given that most hyperkalemia cases in our cohort was detected at or before day 30 (**Supplementary Figure S6**), the first post-initiation measurement is likely to carry the greatest yield. As finerenone uptake expands following its inclusion in the National Reimbursement Drug List, these effectiveness and safety data can inform the forthcoming update to the Chinese finerenone consensus.

### Strengths and limitations

This study has several strengths. It is the first multicenter TTE of the finerenone–empagliflozin combination using Chinese EHR data, addressing a population entirely absent from the CONFIDENCE trial. The TTE framework with explicit time-zero alignment reduces prevalent user bias and prevents the misalignment of eligibility, assignment, and start of follow-up that generates immortal time bias (15, 17, 25). The clone–censor–weight approach, which provides our primary estimate, avoids both the selection bias inherent in naive per-protocol restriction and the grace-period immortal time to which the assignment contrast is exposed (28, 33). PS matching achieved excellent covariate balance (all SMDs <0.10; **Supplementary Figure S2**), and the results were robust across 10 sensitivity analyses (**Supplementary Figure S4**).

Several limitations warrant consideration, and two of them constrain every estimate that we report. First, loss to follow-up was substantial, ranging from 29.6% to 32.7% across arms, and day-180 UACR was missing for 14.7% to 16.5% of patients, as is common in multicenter EHR studies without active participant retention. Loss to follow-up of this magnitude is a recognized threat to internal validity in cohort studies, and whether it produces bias depends on the underlying causal structure rather than on the proportion lost alone (42). Both of our analyses assume that these data are missing at random conditional on the measured covariates: the assignment analysis through multiple imputation and the per-protocol analysis through inverse probability of censoring weights, which correct informative censoring only under that same conditional independence (34). The assumption is not verifiable from the observed data and is questionable if patients who deteriorated, became non-adherent, or transferred care were differentially lost by arm. Second and directly related, we assessed robustness to departures from this assumption in a delta-adjusted tipping-point analysis in which day-180 UACR values for combination-strategy patients without an observed day-180 measurement (*n* = 142) were penalized by a progressively larger increment (43). The combination versus finerenone difference attenuated from −19.6 percentage points at no penalty to −18.1 at 10 percentage points, −16.6 at 20, −15.2 at 30, and −12.2 at 50, and the upper bound of the 95% confidence interval excluded the null at every value examined (**Supplementary Table S9**). No tipping point was reached within a plausible range, so the primary finding is robust to substantial departures from the missing-at-random assumption, although estimates remain conditional on the assumed pattern of departure. Second, mortality ascertainment was limited to in-hospital deaths; without linkage to a national death registry, out-of-hospital deaths were missed. Third, Chinese EHR data present well-documented quality challenges, including non-standardized coding practices and limited cross-institutional linkage (44, 45). Fourth, UACR measurement was available on day 180 in approximately 85% of patients; the missing-at-random assumption underlying our imputation approach may not fully hold. Fifth, treatment was assigned by physicians rather than at random, and confounding by indication is the principal threat to validity. Clinicians who prescribe dual therapy may do so for patients they judge to have more aggressive or more modifiable disease, greater expected adherence, better health literacy, or greater ability to afford and attend monitoring; conversely, monotherapy may be preferred for frailer patients or those judged to be at higher hyperkalemia risk. None of these judgements is recorded in structured EHR fields, and all could plausibly act in the direction of the observed benefit. Although E-value analyses indicate that substantial unmeasured confounding would be required to negate the observed association, E-values quantify only the strength of association such a confounder would need and cannot establish that no such confounder exists. Residual imbalance persisted for DPP-4 inhibitor use (SMD 0.127) and LDL cholesterol (SMD 0.117); neither is a recognized determinant of albuminuria response, but double-adjustment for covariates exceeding an SMD of 0.10 was not undertaken and residual confounding from these variables cannot be excluded (31). Sixth, safety comparisons may be affected by differential ascertainment: patients receiving finerenone are recommended to undergo more frequent potassium and creatinine monitoring than those receiving an SGLT2 inhibitor alone, so biochemical events are more likely to be detected in finerenone-containing arms (46). We were unable to compare per-patient laboratory testing frequency across arms within the present extraction, so the magnitude and direction of this surveillance bias cannot be quantified; it would be expected to inflate apparent hyperkalemia and acute kidney injury rates in the finerenone-containing arms relative to empagliflozin monotherapy. Seventh, hospital site, calendar time of initiation, and monitoring intensity were not included in the propensity score or censoring models; unmeasured site-level and secular differences in prescribing and follow-up may therefore persist. Eighth, reasons for and timing of treatment discontinuation were not systematically recorded, and in the combination arm discontinuation of each agent was captured separately without a structured indicator of whether both agents stopped concurrently. Ninth, the number of patients contributed by each participating hospital was not available in the analytic extraction, so site-level heterogeneity could not be examined. Tenth, the 90-day grace period means that the assignment contrast is not an intention-to-treat effect and retains a component of immortal time; although the per-protocol estimate is free of this bias by construction and the two were concordant, the assignment estimates should not be interpreted as though they came from a randomized comparison. Eleventh, the primary endpoint was change in albuminuria, an accepted but imperfect surrogate: a 30% reduction in albuminuria is associated with a lower risk of subsequent kidney failure and is accepted by regulators as a reasonably likely surrogate in early CKD, yet the trial-level association with hard outcomes is only moderately strong, and a surrogate benefit does not establish long-term cardiorenal protection (10, 11). The 180-day follow-up precludes assessment of kidney failure, cardiovascular events, or mortality. Finally, all patients were managed in three tertiary (grade A) hospitals, 24.3% of eligible patients were not retained by propensity score matching, and the matched population is by construction restricted to the region of covariate overlap. Our findings should therefore be applied to comparable Chinese tertiary-care populations and should not be extrapolated to primary care, community practice, unselected Chinese patients, or non-Chinese settings.

## Conclusions

In this TTE of the CONFIDENCE trial using Chinese multicenter EHR data, combination finerenone and empagliflozin was associated with an approximately 52% reduction in albuminuria at day 180, closely replicating the albuminuria findings of the original trial. Primary inference rests on the per-protocol clone–censor–weight estimate, which is not susceptible to the immortal time bias introduced by the grace period; the assignment contrast, which is not a true intention-to-treat effect, was concordant and is reported as supportive. The association was consistent across prespecified subgroups and robust to multiple sensitivity analyses. Because albuminuria is a surrogate endpoint and follow-up was limited to 180 days, these data replicate only the short-term surrogate effect and do not establish long-term cardiorenal benefit in Chinese patients. Hyperkalemia was common in finerenone-containing arms, and acute kidney injury and symptomatic hypotension were numerically more frequent with combination therapy, so combination therapy warrants structured biochemical monitoring rather than unqualified endorsement. Certainty is further limited by loss to follow-up of nearly one-third, although a delta-adjusted tipping-point analysis showed the primary finding to be robust to substantial departures from the missing-at-random assumption. These findings are applicable to comparable Chinese tertiary-care populations; longer-term studies powered for hard kidney and cardiovascular endpoints and with more complete follow-up are needed before broader recommendations can be made.

## Data Availability

The raw data supporting the conclusions of this article will be made available by the authors, without undue reservation.
