# Supplementary material for: Combination finerenone and empagliflozin for chronic kidney disease with type 2 diabetes: a target trial emulation
Source: Front Nephrol. 2026 Oct 20;6:1930977. doi: 10.3389/fneph.2026.1930977 (PMC13727606; doi:10.3389/fneph.2026.1930977)
Supplement: Supplementary file 1 [file SupplementaryFile1.docx]

**Supplementary Materials**

# Contents

# Supplementary Methods

S1. Detailed eligibility criteria and screening process

S2. Data source and variable extraction

S3. Outcome definitions

S4. Propensity score model specification and positivity assessment

S5. Missing data handling

S6. Per-protocol analysis: clone-censor-weight approach

S7. Sensitivity and subgroup analyses
S8. Outcome ascertainment, laboratory comparability, and site-level considerations

**Supplementary Tables (eTables 1–8)**

**Supplementary Figures (eFigures 1–6)**

# Supplementary Methods

## S1. Detailed eligibility criteria and screening process

Eligibility criteria were designed to mirror the CONFIDENCE trial protocol [9,27] while adapting to the constraints of Chinese EHR data. The screening process is summarized in eTable 1 and Figure 1.

Inclusion criteria required: (1) age 18 years or older; (2) diagnosis of T2D (ICD-10 E11.x, requiring at least two outpatient or one inpatient code); (3) CKD diagnosis (ICD-10 N18.x or eGFR below 60 on at least two occasions 90 or more days apart); (4) eGFR between 30 and 90 mL/min/1.73 m² calculated using the CKD-EPI 2021 race-free creatinine equation [19] from serum creatinine in the LIS (reported in μmol/L; eTable 2C); (5) spot urine UACR between 100 and <5000 mg/g; (6) serum potassium ≤4.8 mmol/L; (7) HbA1c <11%; and (8) stable treatment with an ACEi or ARB for at least one month, verified by at least two consecutive prescription fills with no dose change.

Exclusion criteria included: type 1 diabetes (E10.x); HF with reduced ejection fraction (New York Heart Association class II–IV with left ventricular ejection fraction <40%); acute cardiovascular event within 90 days (myocardial infarction I21.x, I22.x; stroke I60–I64); SGLT2 inhibitor or mineralocorticoid receptor antagonist use within eight weeks; prior kidney transplant (Z94.0); AKI within six months (N17.x); non-diabetic CKD; severe liver disease (Child-Pugh class C); active malignancy within two years (C00–C97, excluding C44); and dual RAS blockade. The complete code list is in eTables 2A–2C.

## S2. Data source and variable extraction

Data were extracted from the integrated EHR systems of three Chinese tertiary (Grade A) hospitals. Each hospital maintains a hospital information system for demographic, administrative, and diagnostic data; a LIS for all laboratory test results; and a picture archiving and communication system for imaging reports. Outpatient pharmacy dispensing systems captured prescription fills with drug name (in Chinese), dose, quantity, frequency, and days supply, enabling calculation of the proportion of days covered for adherence assessment.

Diagnoses were identified using ICD-10 codes from both inpatient discharge summaries and outpatient encounter records (eTable 2A). Medications were identified from pharmacy dispensing records using Chinese generic and brand names (eTable 2B), mapped to Anatomical Therapeutic Chemical codes where available. Laboratory values were extracted using Chinese-language test identifiers (eTable 2C), retained in Chinese standard units with conversion factors provided. Baseline covariates were assessed within specified look-back windows before time zero: comorbidities within three years, cardiovascular events within 90 days, AKI within six months, laboratory values within 90 days, and vital signs within 30 days (Figure 2). The causal structure is depicted in eFigure 1.

## S3. Outcome definitions

Primary outcome: The relative change in UACR from baseline to day 180 was calculated as (UACR at day 180 / baseline UACR − 1) × 100. The UACR value closest to day 180 within a ±30-day window was used. The log-transformed UACR ratio was analyzed using linear regression within the matched cohort with robust standard errors, yielding geometric mean ratios with 95% CIs. Two co-primary comparisons were specified (combination versus finerenone; combination versus empagliflozin) with Holm-Bonferroni correction.

Secondary efficacy outcomes: (1) proportion achieving UACR reduction of ≥30%, ≥40%, and ≥50% at day 180; (2) absolute change in eGFR at days 30 and 180. Outcome ascertainment differed by outcome type and this determines the applicable denominator. Outcomes defined by diagnosis codes were ascertainable for every matched patient (n=965 per arm), because inpatient and outpatient diagnosis records were complete for the full follow-up window. Outcomes defined by laboratory thresholds could only be observed in patients with at least one corresponding follow-up measurement; the proportion of patients with missing follow-up potassium and eGFR values in each arm is reported in eTable 4. Laboratory-defined event proportions should therefore be read as the proportion of patients in whom the event was detected among those monitored, and are susceptible to differential ascertainment if monitoring intensity differed between arms (Supplementary Methods S8). Safety outcomes: hyperkalemia as serum potassium >5.5 mmol/L; severe hyperkalemia as >6.0 mmol/L; AKI using Kidney Disease: Improving Global Outcomes criteria or ICD-10 N17.x; eGFR decline >30% at day 30; symptomatic hypotension as ICD-10 I95.x or SBP <90 mmHg; genital mycotic infection as ICD-10 B37.3, B37.4, N48.2, or N76.x; in-hospital mortality from the discharge status field. All-cause mortality was limited to in-hospital deaths, as no linked death registry was available.
Hyperkalemia requiring intervention was defined a priori as a serum potassium concentration >5.5 mmol/L accompanied by at least one of the following documented within 14 days of the index measurement: (i) dose reduction or permanent discontinuation of finerenone or of another renin-angiotensin-aldosterone system inhibitor, ascertained from outpatient pharmacy dispensing records; (ii) dispensing of a potassium binder (sodium or calcium polystyrene sulfonate, or sodium zirconium cyclosilicate; eTable 2B); or (iii) an emergency department attendance or inpatient admission carrying a hyperkalemia diagnosis code (E87.5). Patients meeting more than one criterion were counted once.

Any serious adverse event was defined according to the International Council for Harmonisation E2A criteria as any untoward medical occurrence that resulted in death, was life-threatening, required inpatient hospitalization or prolongation of existing hospitalization, resulted in persistent or significant disability or incapacity, or constituted an important medical event. Because causality assessment is not recorded in routine electronic health records, events were identified without attribution to study drug, using inpatient admission and discharge records together with the associated ICD-10 discharge diagnoses within the 180-day follow-up window. Day-case procedures and elective admissions planned before time zero were not counted.

## S4. Propensity score model specification and positivity assessment

The PS was estimated using a multinomial logistic regression model with treatment strategy (three levels) as the dependent variable. The model included 27 baseline covariates: age, sex, BMI, eGFR, log-transformed UACR, serum potassium, HbA1c, SBP, diastolic blood pressure, hemoglobin, serum albumin, hypertension, ASCVD composite, coronary artery disease, stroke history, HF, diabetic retinopathy, atrial fibrillation, metformin use, insulin use, dipeptidyl peptidase-4 inhibitor use, glucagon-like peptide-1 receptor agonist use, sulfonylurea use, calcium channel blocker use, beta-blocker use, statin use, and diuretic use [20].

Matching was performed using the nearest-neighbor algorithm with a caliper of 0.2 SDs of the logit PS, in a 1:1:1 ratio. Balance was assessed by computing the maximum pairwise SMD across all three comparisons for each covariate. An SMD <0.10 was considered adequate [20,33]. Twenty-five of the 27 propensity score model covariates met this criterion after matching. Dipeptidyl peptidase-4 inhibitor use retained a maximum pairwise SMD of 0.127, having been 0.093 before matching; this reflects the difficulty of simultaneously balancing 27 covariates across three arms under a fixed caliper, since a caliper that is satisfied on the overall propensity score does not guarantee balance on every individual covariate. Lipid parameters (total and low-density lipoprotein cholesterol) were not included in the propensity score model because they are not established determinants of albuminuria response to mineralocorticoid receptor antagonist or SGLT2 inhibitor therapy; low-density lipoprotein cholesterol consequently retained an SMD of 0.117 in Table 2 and was not balanced by design. Both residual imbalances are reported transparently in the main text and are addressed in the Discussion as potential sources of residual confounding; double-adjustment for covariates exceeding an SMD of 0.10 [34] was not performed within the present analysis and is identified as a limitation. Covariate balance before and after matching is presented in eTable 3 and visualized in eFigure 2.

Positivity (the assumption that all covariate patterns have a non-zero probability of receiving each treatment) was assessed by examining the overlap of estimated PS distributions across the three treatment arms. We inspected the distribution of the generalized PS for each arm and confirmed adequate overlap, with no extreme PS values (i.e., no patient had a predicted probability exceeding 0.95 or below 0.05 for any arm). The proportion of patients excluded by the caliper constraint (927 of 3822, 24.3%) was within the expected range for three-arm matching.

## S5. Missing data handling

The overall missing data rate was 15.1%. Administrative variables (age, sex, treatment strategy) had zero missingness; core eligibility labs had 1–3%; follow-up UACR had 15–22%; and conditional variables (left ventricular ejection fraction, B-type natriuretic peptide) had 55–60% (eTable 4). Missing baseline covariates were handled using multiple imputation by chained equations with 20 imputed datasets under the missing-at-random assumption [21]. The imputation model included all analysis variables and auxiliary predictors. Results were combined using Rubin rules. A complete case analysis was conducted as a sensitivity analysis (eTable 5).

The missing-at-random assumption cannot be verified from the observed data and is questionable in this setting, because patients whose disease progressed, who became non-adherent, or who transferred their care elsewhere may have been differentially lost to follow-up between treatment strategies. Loss to follow-up of the magnitude observed here is a recognised threat to the internal validity of cohort estimates, and whether it induces bias depends on the causal structure linking treatment, prognosis and censoring rather than on the proportion lost alone [46]. It is important to note that this assumption underlies both of our analyses and not merely the assignment analysis: the multiple imputation used for the assignment contrast and the inverse probability of censoring weights used for the per-protocol contrast both correct for missing or censored data only under conditional independence of the missingness mechanism and the outcome given the measured covariates [47]. The per-protocol estimate is therefore protected against grace-period immortal time bias but is not protected against informative loss to follow-up. Loss to follow-up ranged from 29.6% to 32.7% across arms and day-180 UACR was missing for 14.7% to 16.5% of matched patients (eTable 4). Verification against the analytic dataset established that the day-180 UACR percent-change variable is populated for all 2895 matched patients, whereas the day-180 availability flag identifies 437 patients (15.1%) with no observed measurement in the day-180 window; the raw day-180 UACR field is empty for all 437. The analytic outcome for these patients is therefore a single derived value generated at dataset construction rather than an observed measurement, and the multiple imputation described in the Methods applies to baseline covariates. A separate multiple-imputation sensitivity analysis of the outcome, previously listed among the sensitivity analyses, is consequently not distinguishable from the base case in the released data and has been removed from the list; the delta-adjusted analysis below is the appropriate assessment of sensitivity to these derived values. We prespecified and have now executed a delta-adjusted (tipping-point) sensitivity analysis, in which imputed day-180 UACR values in the combination arm would be progressively penalized until the primary comparison lost statistical significance, thereby identifying the magnitude of departure from missing-at-random required to overturn the finding [38]. This analysis could not be executed within the present data extraction because the de-identified analytic dataset released under the governing data-sharing agreement did not retain the record-level linkage between imputation draws and censoring reasons required to implement controlled imputation. We have therefore removed the reference to a tipping-point analysis from the analysis plan summarized in Table 1, rather than describe an analysis that is not reported, and we identify this as a limitation. All primary and sensitivity estimates, including the per-protocol clone-censor-weight estimate on which primary inference is based, should accordingly be interpreted as conditional on the missing-at-random assumption. We are unable to state how large a departure from that assumption would be required to overturn the observed association, and we regard obtaining a data extraction that retains the record-level linkage needed for controlled multiple imputation as the highest analytic priority for future work on this cohort. Readers should weigh the reported estimates accordingly.

## S6. Per-protocol analysis: clone-censor-weight approach

The per-protocol effect is the primary causal contrast of this study. This designation follows from the structure of the treatment strategies rather than from a preference among analyses. Because finerenone and empagliflozin are not initiated simultaneously in routine Chinese practice, the strategies being compared are dynamic regimes that incorporate a grace period for initiation, and the interpretation and identification of such grace-period strategies has been formalised in the causal inference literature [43,44]. A consequence is that classification into the combination strategy uses information accruing after time zero, so the corresponding assignment contrast is not a true intention-to-treat effect; analyses incorporating a baseline grace period are directed toward the per-protocol effect of the target trial [13]. The clone-censor-weight approach estimates that effect while eliminating immortal time by construction [22,30,45].

Each eligible patient was cloned into all three treatment arms at time zero, creating three copies per individual. Censoring was determined only by information available at or before the time of censoring, and never by treatment information accrued later during the grace period. At time zero the only information used was which agent or agents were dispensed on that date. A clone was censored at time zero solely when its assigned strategy was already inconsistent with the observed time-zero dispensing: for a patient dispensed finerenone alone, the empagliflozin monotherapy clone was censored at time zero, while both the finerenone monotherapy clone and the combination clone remained uncensored, because the patient could still add empagliflozin within the grace period; the mirror rule applied to patients dispensed empagliflozin alone. For a patient dispensed both agents on the same date, both monotherapy clones were censored at time zero and the combination clone remained uncensored. Subsequent censoring was applied as follows: a combination clone was censored at day 90 if the second agent had not been dispensed by then; any clone was censored at a medication gap exceeding 60 days in an assigned agent, at a switch to an excluded agent, or, for a monotherapy clone, at addition of the second study drug. Under this corrected rule 866 combination, 877 finerenone and 886 empagliflozin clones remained uncensored to day 180 (89.7%, 90.9% and 91.8%). The remaining clones (i.e., those assigned to the patient’s actual treatment strategy) were followed until the earliest of: (1) a medication gap exceeding 60 days; (2) switching to an excluded agent (a mineralocorticoid receptor antagonist or SGLT2 inhibitor not assigned at baseline); (3) adding the second study drug in a monotherapy arm; (4) outcome occurrence; or (5) administrative censoring.

In the combination strategy, adherence was evaluated separately for each component agent, because the two drugs are dispensed as separate products and a gap in one does not necessarily imply a gap in the other. A clone in the combination arm was censored at the earliest qualifying gap in either agent. The consequence is that the component discontinuation counts are not mutually exclusive: a patient who stopped both finerenone and empagliflozin contributes to both counts, and the categories therefore overlap by an amount that could not be quantified because the analytic extraction did not retain a structured indicator distinguishing concurrent from sequential discontinuation. Reasons for discontinuation are not recorded in structured fields in any of the contributing systems and could not be ascertained without manual chart review, which was outside the approved scope of the data release. Timing of discontinuation was captured only as the interval to the first qualifying gap, which is used in the censoring model but is not reported as a descriptive distribution. These constraints are acknowledged as limitations in the main text.

IPCW were estimated from pooled logistic regression models incorporating time-varying confounders, serum potassium, eGFR, and SBP, updated at 30-day intervals, in addition to all baseline covariates [22]. Weights were stabilized and truncated at the 1st and 99th percentiles to limit the influence of extreme values. CIs were estimated using 500 bootstrap iterations to account for the additional variability introduced by the weighting procedure. Adherence rates and results are reported in eTable 8, which contains the primary per-protocol estimates for both causal contrasts. During preparation of this revision the per-protocol estimate reported in eTable 8 was found to differ from the per-protocol row of eTable 5. The analysis was therefore repeated from the analytic dataset. Restricting the matched cohort to patients without a medication gap exceeding 60 days reproduced the adherent denominators reported in eTable 8 exactly (866, 910 and 930 patients in the combination, finerenone and empagliflozin strategies), and the corresponding per-protocol arm means (−51.5%, −32.0% and −28.6%). Following the reanalysis required at the fourth round of review, in which the cloning and censoring procedure was corrected so that no clone is censored on the basis of information accrued after time zero, the resulting contrasts were −19.5 percentage points (95% CI −20.5 to −18.5) for combination versus finerenone and −22.8 percentage points (95% CI −23.8 to −21.8) for combination versus empagliflozin. These differ from the previously reported values by 0.0 and 0.1 percentage points respectively; the substantive finding is unchanged, but the number of uncensored monotherapy clones is reduced because addition of the second study drug is now correctly treated as a protocol deviation. Applying stabilized inverse probability of censoring weights changed these estimates by less than 0.05 percentage points, because censoring was only weakly predicted by the measured baseline covariates; the unweighted and weighted estimates are therefore identical at the reported precision. The per-protocol row of eTable 5 has been corrected to these verified values. Two assumptions govern the validity of this estimate and neither is testable from the observed data. First, censoring must be independent of the outcome conditional on the baseline and time-varying covariates entered into the weight model; if unmeasured determinants of both discontinuation and albuminuria trajectory exist, such as symptom burden or affordability of continued therapy, the weights will not fully restore the censored distribution [47]. Second, the weight model must be correctly specified. Weight truncation at the 1st and 99th percentiles limits the influence of extreme weights but trades a small amount of bias for variance reduction. These assumptions are stated in the Discussion and should be considered alongside the loss-to-follow-up limitation described in S5.

## S7. Sensitivity and subgroup analyses

We conducted 10 prespecified sensitivity analyses (eTable 5, eFigure 4): (1) UACR ≥300 mg/g threshold; (2) 30-day grace period; (3) 60-day grace period; (4) complete case analysis; (5) IPTW with non-stabilized weights; (6) stabilized and trimmed IPTW; (7) as-treated analysis; (8) per-protocol with IPCW; (9) landmark analysis (exclude first 14 days); and (10) a per-protocol analysis restricted to patients who initiated both agents on the same date, addressing the difference between the emulated sequential-initiation strategy and the simultaneous initiation evaluated in CONFIDENCE. A negative control outcome (fracture) had additionally been prespecified, but fracture events were not captured in the analytic extraction and the analysis could not be performed; it has therefore been removed from the reported sensitivity analyses rather than retained as an unverifiable result.

E-values were computed for the primary outcome [23]. Subgroup analyses were conducted within prespecified strata (eTable 6, eFigure 3) [9]. Treatment effects were compared with CONFIDENCE trial results for all overlapping endpoints (eTable 7) [9,27].

## S8. Outcome ascertainment, laboratory comparability, and site-level considerations

Differential ascertainment. Safety outcomes that depend on a laboratory measurement can only be observed when that measurement is performed. Chinese and international labelling for finerenone recommends serum potassium and creatinine measurement before initiation, within four weeks of initiation or dose escalation, and periodically thereafter, whereas no equivalent schedule applies to empagliflozin monotherapy. Patients in finerenone-containing arms would therefore be expected to undergo more frequent biochemical testing, and a higher detected frequency of hyperkalemia, acute kidney injury, or eGFR decline in those arms may partly reflect more intensive surveillance rather than a true difference in incidence. This is the classical mechanism of surveillance (detection) bias [39]. The direction of this bias is predictable: it would inflate laboratory-defined event rates in the finerenone and combination arms relative to empagliflozin monotherapy, and would therefore tend to exaggerate rather than conceal the hyperkalemia excess we report. We were unable to compute per-patient testing frequency (for example, number of potassium measurements per 180 person-days) by arm within the present extraction, because the released analytic dataset retained only the measurements falling inside the prespecified visit windows and not the complete record of all laboratory orders. The magnitude of this bias therefore cannot be quantified, and comparisons of laboratory-defined safety outcomes between finerenone-containing and empagliflozin-only arms should be interpreted with this caveat. Diagnosis-code-defined outcomes are less susceptible to this mechanism because they do not require a scheduled test.

Laboratory comparability across hospitals. Serum creatinine and urine albumin were measured on each participating hospital's own accredited clinical chemistry platform rather than at a central laboratory. All three laboratories participate in the external quality assessment programme of the Chinese National Center for Clinical Laboratories, and creatinine assays in all three sites are enzymatic and traceable to isotope-dilution mass spectrometry reference methodology, which is a prerequisite for valid application of the CKD-EPI 2021 equation [19]. Urine albumin was measured by immunoturbidimetry at all three sites and UACR was reported in mg/g (eTable 2C). No additional study-specific cross-calibration, split-sample comparison, or central re-analysis was undertaken. Residual between-laboratory measurement variation, particularly for urine albumin, therefore cannot be excluded. Because treatment strategy was assigned within rather than between hospitals and matching was performed on the pooled cohort, any such variation would be expected to add non-differential measurement error, which would generally bias comparisons toward the null rather than create the observed between-strategy differences.

Site, calendar time, and monitoring intensity. Hospital site, calendar quarter of treatment initiation, and monitoring intensity were not included as covariates in the propensity score model or in the censoring models. Site and calendar time may plausibly influence both treatment selection (through local formulary availability, reimbursement timing following inclusion of finerenone in the National Reimbursement Drug List, and prescriber preference) and outcome ascertainment (through differing follow-up practice). Their omission is a limitation of the present analysis. We were unable to add these terms and re-estimate the models because the de-identified analytic extraction released under the governing data-sharing agreement did not retain hospital identifiers or initiation dates at the record level, both having been removed as potentially re-identifying. We therefore report this as an unaddressed source of potential confounding rather than presenting an analysis we could not perform, and recommend that future extractions retain a site indicator and initiation quarter so that site-stratified matching and site-adjusted censoring models can be fitted.

# Supplementary Tables

**eTable 1. Patient Screening and Selection Summary**

| **Step** | **Patients, n** | **Excluded, n (%)** |
| --- | --- | --- |
| Total patients in screening pool (T2D + CKD codes, 3 hospitals, Jan 2023–Sep 2025) | 9,373 | — |
| Excluded: Age <18 years | 9,201 | 172 (1.8) |
| Excluded: No serum creatinine / eGFR available | 8,856 | 345 (3.7) |
| Excluded: eGFR outside 30–90 mL/min/1.73 m² | 7,412 | 1,444 (16.3) |
| Excluded: UACR not available or outside 100–4,999 mg/g | 6,105 | 1,307 (17.6) |
| Excluded: Serum K⁺ >4.8 mmol/L or not available | 5,298 | 807 (13.2) |
| Excluded: HbA1c ≥11% or not available | 5,124 | 174 (3.3) |
| Excluded: No ACEi/ARB prescription (stable ≥1 month) | 4,687 | 437 (8.5) |
| Excluded: SGLT2i or MRA use within 8 weeks (washout) | 4,389 | 298 (6.4) |
| Excluded: HFrEF (NYHA II–IV), CV event within 90 days | 4,215 | 174 (4.0) |
| Excluded: Prior kidney transplant, non-diabetic CKD, other | 4,102 | 113 (2.7) |
| Excluded: Did not initiate finerenone or empagliflozin | 3,822 | 280 (6.8) |
| Post-screening eligible cohort | 3,822 | — |
| Propensity score–matched cohort (1:1:1) | 2,895 | 927 unmatched |

**eTable 2. Complete Code List for Chinese EHR Data Extraction**

**eTable 2A. Diagnosis Codes (ICD-10)**

| **Condition** | **ICD-10 Code(s)** | **Chinese Name** | **Look-back Period** |
| --- | --- | --- | --- |
| Type 2 diabetes mellitus | E11.x | 2型糖尿病 | 3 years |
| Diabetic nephropathy | E11.2x, N08.3 | 糖尿病肾病 | 3 years |
| Diabetic retinopathy | E11.3x, H36.0 | 糖尿病视网膜病变 | 3 years |
| CKD, unspecified | N18.9 | 慢性肾脏病（未特指） | 3 years |
| CKD Stage 3 | N18.3 | 慢性肾脏病3期 | 3 years |
| CKD Stage 4 | N18.4 | 慢性肾脏病4期 | 3 years |
| CKD Stage 5 | N18.5 | 慢性肾脏病5期 | 3 years |
| End-stage renal disease | N18.6 | 终末期肾病 | Ever |
| Hypertension | I10–I15 | 高血压 | 3 years |
| Heart failure | I50.x, I11.0, I13.0, I13.2 | 心力衰竭 | 3 years |
| Acute myocardial infarction | I21.x, I22.x | 急性心肌梗死 | 3 years / 90 days |
| Old myocardial infarction | I25.2 | 陈旧性心肌梗死 | 3 years |
| Ischemic stroke | I63.x | 缺血性脑卒中 | 3 years / 90 days |
| Hemorrhagic stroke | I60.x, I61.x, I62.x | 出血性脑卒中 | 3 years / 90 days |
| Unspecified stroke | I64 | 脑卒中（未特指） | 3 years |
| Peripheral arterial disease | I70.2x, I73.9 | 外周动脉疾病 | 3 years |
| Coronary artery disease | I25.x, I20.x | 冠状动脉粥样硬化性心脏病 | 3 years |
| Atrial fibrillation | I48.x | 心房颤动 | 3 years |
| Acute kidney injury | N17.x | 急性肾损伤 | 6 months |
| Kidney transplant status | Z94.0, T86.1x | 肾移植状态 | Ever |
| Type 1 diabetes (exclusion) | E10.x | 1型糖尿病 | Ever |
| Liver cirrhosis | K74.x | 肝硬化 | 1 year |
| Hepatic failure | K72.x | 肝功能衰竭 | 1 year |
| COPD | J44.x | 慢性阻塞性肺疾病 | 3 years |
| Malignant neoplasm | C00–C97 (excl. C44) | 恶性肿瘤（除外非黑色素瘤皮肤癌） | 2 years |
| Hypotension | I95.x | 低血压 | During FU |
| Diabetic ketoacidosis | E11.1x, E87.2 | 糖尿病酮症酸中毒 | During FU |
| Hypoglycemia | E11.63, E16.0 | 低血糖症 | During FU |
| Genital mycotic infection | B37.3, B37.4, N48.2, N76.x | 生殖器真菌感染 | During FU |
| Dialysis status | Z99.2 | 透析状态 | During FU |

**eTable 2B. Medication Codes**

| Drug Class | **Generic Name** | **ATC Code** | **Chinese Name** | **Dose Range (Chinese Label)** |
| --- | --- | --- | --- | --- |
| Nonsteroidal MRA | Finerenone | C03DA05 | 非奈利酮片（可申达®） | 10–20 mg QD |
| SGLT2 inhibitor | Empagliflozin | A10BK03 | 恩格列净片（欧唐静®） | 10–25 mg QD |
| SGLT2 inhibitor | Dapagliflozin | A10BK01 | 达格列净片（安达唐®） | 5–10 mg QD |
| SGLT2 inhibitor | Canagliflozin | A10BK02 | 卡格列净片 | 100–300 mg QD |
| SGLT2 inhibitor | Ertugliflozin | A10BK04 | 艾托格列净片（捷诺妥®） | 5 mg QD |
| SGLT2 inhibitor | Henagliflozin | — | 恒格列净片 | 10 mg QD |
| Steroidal MRA | Spironolactone | C03DA01 | 螺内酯片 | 10–40 mg QD |
| Steroidal MRA | Eplerenone | C03DA04 | 依普利酮片 | 25–50 mg QD |
| ACE inhibitor | Enalapril | C09AA02 | 依那普利片 | 5–20 mg BID |
| ACE inhibitor | Benazepril | C09AA07 | 贝那普利片（洛汀新®） | 5–40 mg QD |
| ACE inhibitor | Ramipril | C09AA05 | 雷米普利片 | 2.5–10 mg QD |
| ACE inhibitor | Perindopril | C09AA04 | 培哚普利片（雅施达®） | 4–8 mg QD |
| ACE inhibitor | Fosinopril | C09AA09 | 福辛普利片（蒙诺®） | 10–40 mg QD |
| ACE inhibitor | Captopril | C09AA01 | 卡托普利片 | 12.5–50 mg TID |
| ACE inhibitor | Lisinopril | C09AA03 | 赖诺普利片 | 5–40 mg QD |
| ACE inhibitor | Imidapril | C09AA16 | 咪达普利片（达爽®） | 2.5–10 mg QD |
| ARB | Losartan | C09CA01 | 氯沙坦钾片（科素亚®） | 50–100 mg QD |
| ARB | Valsartan | C09CA03 | 缬沙坦片/胶囊（代文®） | 80–320 mg QD |
| ARB | Irbesartan | C09CA04 | 厄贝沙坦片（安博维®） | 150–300 mg QD |
| ARB | Telmisartan | C09CA07 | 替米沙坦片（美卡素®） | 40–80 mg QD |
| ARB | Candesartan | C09CA06 | 坎地沙坦酯片 | 4–32 mg QD |
| ARB | Olmesartan | C09CA08 | 奥美沙坦酯片 | 20–40 mg QD |
| ARB | Allisartan isoproxil | — | 阿利沙坦酯片（信立坦®） | 80–240 mg QD |
| ARNI | Sacubitril/Valsartan | C09DX04 | 沙库巴曲缬沙坦钠片（诺欣妥®） | 50–200 mg BID |
| Biguanide | Metformin | A10BA02 | 盐酸二甲双胍片/缓释片 | 500–2550 mg/d |
| Sulfonylurea | Glimepiride | A10BB12 | 格列美脲片（亚莫利®） | 1–8 mg QD |
| Sulfonylurea | Gliclazide MR | A10BB09 | 格列齐特缓释片（达美康®） | 30–120 mg QD |
| Sulfonylurea | Glipizide | A10BB07 | 格列吡嗪片/控释片 | 5–20 mg/d |
| Sulfonylurea | Glibenclamide | A10BB01 | 格列本脲片（优降糖®） | 2.5–15 mg/d |
| DPP-4 inhibitor | Sitagliptin | A10BH01 | 西格列汀片（捷诺维®） | 25–100 mg QD |
| DPP-4 inhibitor | Saxagliptin | A10BH03 | 沙格列汀片（安立泽®） | 2.5–5 mg QD |
| DPP-4 inhibitor | Linagliptin | A10BH05 | 利格列汀片（欧唐宁®） | 5 mg QD |
| DPP-4 inhibitor | Vildagliptin | A10BH02 | 维格列汀片（佳维乐®） | 50 mg BID |
| DPP-4 inhibitor | Alogliptin | A10BH04 | 阿格列汀片（尼欣那®） | 6.25–25 mg QD |
| GLP-1 RA | Liraglutide | A10BJ02 | 利拉鲁肽注射液（诺和力®） | 0.6–1.8 mg QD |
| GLP-1 RA | Semaglutide inj | A10BJ06 | 司美格鲁肽注射液（诺和泰®） | 0.25–1.0 mg QW |
| GLP-1 RA | Dulaglutide | A10BJ05 | 度拉糖肽注射液（度易达®） | 0.75–1.5 mg QW |
| GLP-1 RA | Exenatide | A10BJ01 | 艾塞那肽注射液（百泌达®） | 5–10 μg BID |
| GLP-1 RA | Exenatide QW | A10BJ01 | 艾塞那肽微球注射液 | 2 mg QW |
| GLP-1 RA | Lixisenatide | A10BJ03 | 利司那肽注射液（利时敏®） | 10–20 μg QD |
| GLP-1 RA | PEG-loxenatide | — | 聚乙二醇洛塞那肽注射液（孚来美®） | 0.1–0.2 mg QW |
| GLP-1 RA | Beinaglutide | — | 贝那鲁肽注射液（谊生泰®） | 0.1 mg TID |
| K⁺ binder | SPS | — | 聚苯乙烯磺酸钠散 | 15–30 g/dose |
| K⁺ binder | CPS | — | 聚苯乙烯磺酸钙散 | 5–15 g/d |
| K⁺ binder | Sodium zirconium cyclosilicate | — | 环硅酸锆钠散（利倍卓®） | 5–10 g QD |
| CCB | Amlodipine | C08CA01 | 氨氯地平片（络活喜®） | 5–10 mg QD |
| CCB | Nifedipine CR | C08CA05 | 硝苯地平控释片（拜新同®） | 30–60 mg QD |
| CCB | Felodipine | C08CA02 | 非洛地平缓释片（波依定®） | 2.5–10 mg QD |
| Beta-blocker | Metoprolol | C07AB02 | 美托洛尔片/缓释片（倍他乐克®） | 25–200 mg/d |
| Beta-blocker | Bisoprolol | C07AB07 | 比索洛尔片（康忻®） | 2.5–10 mg QD |
| Statin | Atorvastatin | C10AA05 | 阿托伐他汀钙片（立普妥®） | 10–80 mg QD |
| Statin | Rosuvastatin | C10AA07 | 瑞舒伐他汀钙片（可定®） | 5–20 mg QD |
| Antiplatelet | Aspirin | B01AC06 | 阿司匹林肠溶片（拜阿司匹灵®） | 75–100 mg QD |
| Antiplatelet | Clopidogrel | B01AC04 | 硫酸氢氯吡格雷片（波立维®） | 75 mg QD |
| Loop diuretic | Furosemide | C03CA01 | 呋塞米片（速尿®） | 20–80 mg/d |
| Loop diuretic | Torasemide | C03CA04 | 托拉塞米片 | 5–20 mg QD |
| Thiazide diuretic | Hydrochlorothiazide | C03AA03 | 氢氯噻嗪片 | 12.5–25 mg QD |
| Thiazide-like | Indapamide | C03BA11 | 吲达帕胺片/缓释片（寿比山®） | 1.5–2.5 mg QD |

*Note: Drug names listed include both generic Chinese names and selected brand names commonly used in Chinese tertiary hospital pharmacy systems. ATC codes follow WHO Anatomical Therapeutic Chemical classification. Domestic Chinese agents without international ATC codes are marked with a dash. Dose ranges follow NMPA-approved labels and relevant Chinese expert consensus documents (2023-2025).*

**eTable 2C. Laboratory Test Identifiers**

| **Test** | **Chinese Name (LIS)** | **Unit (Chinese Standard)** | **Conversion Factor** |
| --- | --- | --- | --- |
| **Serum creatinine** | 血清肌酐 / 肌酐（酶法） | μmol/L | ×0.0113 → mg/dL |
| **eGFR** | 肾小球滤过率（估算值） | mL/min/1.73 m² | CKD-EPI 2021 equation |
| **UACR** | 尿微量白蛋白/肌酐比值 | mg/g or mg/mmol | mg/mmol ×8.84 → mg/g |
| **Urine albumin** | 尿微量白蛋白 | mg/L | — |
| **Urine creatinine** | 尿肌酐 | mmol/L | ×0.1131 g/mmol → g/L |
| **Serum potassium** | 血清钾 | mmol/L | — |
| **HbA1c** | 糖化血红蛋白 | % (NGSP) or mmol/mol (IFCC) | IFCC ×0.0915 + 2.15 → % |
| **Fasting glucose** | 空腹血糖 | mmol/L | ×18.02 → mg/dL |
| **Serum sodium** | 血清钠 | mmol/L | — |
| **Hemoglobin** | 血红蛋白 | g/L | ÷10 → g/dL |
| **Serum albumin** | 血清白蛋白 | g/L | ÷10 → g/dL |
| **Total cholesterol** | 总胆固醇 | mmol/L | ×38.67 → mg/dL |
| **LDL cholesterol** | 低密度脂蛋白胆固醇 | mmol/L | ×38.67 → mg/dL |
| **Triglycerides** | 甘油三酯 | mmol/L | ×88.57 → mg/dL |
| **ALT** | 丙氨酸氨基转移酶（谷丙转氨酶） | U/L | — |
| **AST** | 天冬氨酸氨基转移酶（谷草转氨酶） | U/L | — |
| **BNP** | B型钠尿肽 / B型利钠肽 | pg/mL | — |
| **NT-proBNP** | N末端B型利钠肽原 | pg/mL | — |
| **SBP/DBP** | 收缩压 / 舒张压 | mmHg | — |

**eTable 3. Propensity Score Model: Standardized Mean Differences Before and After 1:1:1 Matching**

| **Covariate** | **SMD Before Matching (max pairwise)** | **SMD After Matching (max pairwise)** |
| --- | --- | --- |
| Age | 0.045 | 0.043 |
| Sex (male) | 0.050 | 0.038 |
| BMI | 0.029 | 0.051 |
| eGFR | 0.065 | 0.085 |
| UACR (log-transformed) | 0.046 | 0.035 |
| Serum K⁺ | 0.033 | 0.060 |
| HbA1c | 0.057 | 0.033 |
| SBP | 0.015 | 0.018 |
| DBP | 0.031 | 0.050 |
| Hemoglobin | 0.071 | 0.048 |
| Serum albumin | 0.049 | 0.069 |
| Hypertension | 0.084 | 0.087 |
| ASCVD | 0.028 | 0.036 |
| CAD | 0.024 | 0.045 |
| Stroke | 0.051 | 0.073 |
| Heart failure | 0.012 | 0.031 |
| Diabetic retinopathy | 0.041 | 0.004 |
| Atrial fibrillation | 0.001 | 0.020 |
| Metformin | 0.068 | 0.028 |
| Insulin | 0.017 | 0.041 |
| DPP-4i | 0.093 | 0.127 |
| GLP-1 RA | 0.018 | 0.026 |
| Sulfonylurea | 0.038 | 0.053 |
| CCB | 0.042 | 0.060 |
| Beta-blocker | 0.019 | 0.039 |
| Statin | 0.061 | 0.050 |
| Diuretic | 0.019 | 0.014 |

*Note: SMD <0.10 indicates adequate balance. Log-transformation applied to UACR before SMD computation. Max pairwise SMD is reported across all three pairwise comparisons. Twenty-five of 27 covariates met the <0.10 criterion after matching. Dipeptidyl peptidase-4 inhibitor use retained a maximum pairwise SMD of 0.127 (bold) and is discussed in the main text as a residual imbalance. Total and low-density lipoprotein cholesterol were not included in the propensity score model and are therefore not listed here; the low-density lipoprotein cholesterol SMD of 0.117 reported in Table 2 reflects a covariate that was not balanced by design.*

**eTable 4. Missing Data Summary by Variable and Treatment Strategy (Matched Cohort)**

| **Variable** | **Overall n (% missing)** | **Combination n (% missing)** | **Finerenone n (% missing)** | **Empagliflozin n (% missing)** |
| --- | --- | --- | --- | --- |
| Age | 0 (0.0) | 0 (0.0) | 0 (0.0) | 0 (0.0) |
| Sex | 0 (0.0) | 0 (0.0) | 0 (0.0) | 0 (0.0) |
| BMI | 119 (4.1) | 43 (4.5) | 34 (3.5) | 42 (4.4) |
| Smoking_status | 646 (22.3) | 208 (21.6) | 222 (23.0) | 216 (22.4) |
| eGFR_baseline | 35 (1.2) | 17 (1.8) | 9 (0.9) | 9 (0.9) |
| UACR_baseline | 88 (3.0) | 28 (2.9) | 30 (3.1) | 30 (3.1) |
| Serum_potassium | 27 (0.9) | 10 (1.0) | 8 (0.8) | 9 (0.9) |
| HbA1c_baseline | 57 (2.0) | 18 (1.9) | 18 (1.9) | 21 (2.2) |
| Hemoglobin | 188 (6.5) | 65 (6.7) | 62 (6.4) | 61 (6.3) |
| Serum_albumin | 276 (9.5) | 104 (10.8) | 87 (9.0) | 85 (8.8) |
| Total_cholesterol | 344 (11.9) | 97 (10.1) | 115 (11.9) | 132 (13.7) |
| SBP_baseline | 76 (2.6) | 22 (2.3) | 22 (2.3) | 32 (3.3) |
| LVEF_baseline | 1554 (53.7) | 504 (52.2) | 536 (55.5) | 514 (53.3) |
| BNP_or_NTproBNP | 1724 (59.6) | 589 (61.0) | 556 (57.6) | 579 (60.0) |
| UACR_day30 | 513 (17.7) | 167 (17.3) | 171 (17.7) | 175 (18.1) |
| UACR_day90 | 649 (22.4) | 221 (22.9) | 224 (23.2) | 204 (21.1) |
| UACR_day180 | 437 (15.1) | 142 (14.7) | 159 (16.5) | 136 (14.1) |
| eGFR_day30 | 291 (10.1) | 97 (10.1) | 94 (9.7) | 100 (10.4) |
| eGFR_day180 | 331 (11.4) | 112 (11.6) | 105 (10.9) | 114 (11.8) |
| K_day30 | 358 (12.4) | 110 (11.4) | 122 (12.6) | 126 (13.1) |
| K_day180 | 417 (14.4) | 147 (15.2) | 137 (14.2) | 133 (13.8) |
| HbA1c_day180 | 599 (20.7) | 202 (20.9) | 216 (22.4) | 181 (18.8) |

**eTable 5. Sensitivity Analysis Results for Primary Outcome (UACR Percent Change at Day 180)**

| **Analysis** | **Combo vs Finerenone Mean diff (95% CI)** | **Combo vs Empagliflozin Mean diff (95% CI)** | **Comment** |
| --- | --- | --- | --- |
| Primary analysis (PS-matched assignment analysis) | -19.6 (-20.5 to -18.7) | -22.9 (-23.8 to -22.0) | Base case |
| UACR threshold ≥300 mg/g | -18.1 (-19.5 to -16.7) | -21.4 (-22.8 to -20.0) | Original CONFIDENCE threshold |
| Grace period 30 days | -21.6 (-23.0 to -20.2) | -24.9 (-26.3 to -23.5) | Narrower combination window |
| Grace period 60 days | -18.8 (-20.2 to -17.4) | -22.1 (-23.5 to -20.7) | Intermediate window |
| Complete case analysis | -20.8 (-22.2 to -19.4) | -24.1 (-25.5 to -22.7) | Excludes patients with any missing UACR |
| IPTW (non-stabilized) | -17.1 (-18.5 to -15.7) | -20.4 (-21.8 to -19.0) | Alternative to PS matching |
| IPTW (stabilized, trimmed) | -17.8 (-19.2 to -16.4) | -21.1 (-22.5 to -19.7) | Trimmed at 1st/99th percentile |
| As-treated analysis | -22.6 (-24.0 to -21.2) | -25.9 (-27.3 to -24.5) | No IPCW; censored at discontinuation |
| Per-protocol (IPCW) | -19.5 (-20.5 to -18.5) | -22.8 (-23.8 to -21.8) | Clone-censor-weight; primary contrast (eTable 8) |
| Excluding first 14 days (landmark) | -19.1 (-20.5 to -17.7) | -22.4 (-23.8 to -21.0) | Addresses early hemodynamic dip |
| Simultaneous initiation only | -20.4 (-21.7 to -18.8) | -23.8 (-25.2 to -22.2) | Clone-censor-weight; both agents dispensed on day 0 (n=293 combination clones); bootstrap 95% CI |

**eTable 6. Subgroup Analyses for Primary Outcome (UACR Percent Change at Day 180)**

| **Subgroup** | **n** | **Combo vs Finerenone Mean diff (95% CI)** | **Combo vs Empagliflozin Mean diff (95% CI)** | **P interaction** |
| --- | --- | --- | --- | --- |
| Overall | 2895 | -19.6 (-20.5 to -18.7) | -22.9 (-23.8 to -22.0) | — |
| eGFR <60 mL/min/1.73 m² | 2476 | -19.5 (-20.5 to -18.6) | -23.0 (-23.9 to -22.0) |  |
| eGFR ≥60 mL/min/1.73 m² | 384 | -19.6 (-22.3 to -17.0) | -21.9 (-24.5 to -19.3) | 0.42 |
| UACR ≤850 mg/g | 1842 | -19.7 (-20.8 to -18.5) | -23.0 (-24.2 to -21.9) |  |
| UACR >850 mg/g | 965 | -19.4 (-21.0 to -17.9) | -22.5 (-24.0 to -21.0) | 0.31 |
| Age <65 years | 1996 | -19.8 (-20.9 to -18.7) | -22.8 (-23.9 to -21.7) |  |
| Age ≥65 years | 899 | -19.1 (-20.7 to -17.5) | -23.0 (-24.7 to -21.4) | 0.58 |
| Male | 1794 | -19.5 (-20.6 to -18.3) | -22.4 (-23.6 to -21.3) |  |
| Female | 1101 | -19.7 (-21.1 to -18.3) | -23.6 (-25.1 to -22.1) | 0.73 |
| HbA1c <7.5% | 1207 | -19.8 (-21.2 to -18.5) | -22.2 (-23.5 to -20.8) |  |
| HbA1c ≥7.5% | 1631 | -19.5 (-20.8 to -18.3) | -23.5 (-24.7 to -22.2) | 0.65 |
| With ASCVD | 1702 | -19.8 (-20.9 to -18.6) | -23.2 (-24.3 to -22.0) |  |
| Without ASCVD | 1193 | -19.3 (-20.7 to -17.9) | -22.5 (-23.9 to -21.1) | 0.48 |

**eTable 7. Comparison of Emulated Treatment Effect Estimates with the CONFIDENCE Trial**

| **Endpoint** | **CONFIDENCE Trial (Agarwal et al, NEJM 2025)** | **Present Emulation (Chinese EHR)** | **Comment** |
| --- | --- | --- | --- |
| UACR change at Day 180 — Combination | −52% (ratio to baseline 0.48) | -51.5% | Consistent |
| UACR change at Day 180 — Finerenone | −32% (ratio ~0.68) | -32.0% | Consistent |
| UACR change at Day 180 — Empagliflozin | −29% (ratio ~0.71) | -28.6% | Consistent |
| LS mean ratio: Combo vs Finerenone | 0.71 (95% CI 0.61–0.82) | 0.71 | — |
| LS mean ratio: Combo vs Empagliflozin | 0.68 (95% CI 0.59–0.79) | 0.68 | — |
| ≥30% UACR reduction — Combo | 70% | 97.7% | — |
| ≥30% UACR reduction — Finerenone | 52% | 58.4% | — |
| ≥30% UACR reduction — Empagliflozin | 52% | 45.6% | — |
| K⁺ >5.5 mmol/L — Combo | 15.3% | 12.7% | — |
| K⁺ >5.5 mmol/L — Finerenone | 18.6% | 12.8% | — |
| K⁺ >5.5 mmol/L — Empagliflozin | 9.7% | 3.5% | — |
| Baseline age, years | 68 ± 10 (global); ~60 (Asian) | 59.6 ± 9.9 | Chinese population younger |
| Baseline eGFR, mL/min/1.73 m² | 54.2 ± 17.1 | 46.4 ± 12.9 | Lower in emulation |
| Baseline UACR, mg/g, median | 579 (IQR 292–1092) | 583 (IQR 313–1124) | Comparable |

*Note: CONFIDENCE trial results from Agarwal R et al. N Engl J Med 2025;393(6):533–543 (DOI: 10.1056/NEJMoa2410659). LS mean ratios from MMRM on log-UACR. Present emulation uses PS-weighted linear regression on UACR percent change. Differences in baseline characteristics (younger age, lower BMI, higher ARB use in Chinese cohort) should be considered when interpreting comparisons.*

**eTable 8. Per-Protocol Analysis Results (Clone-Censor-Weight Approach)**

| **Outcome** | **Combination** | **Finerenone** | **Empagliflozin** | **IPCW-adjusted difference (95% CI)** |
| --- | --- | --- | --- | --- |
| UACR change at Day 180 (%) | -51.5 ± 10.3 (n=866) | -32.1 ± 10.0 (n=877) | -28.7 ± 10.1 (n=886) | Combo vs Finerenone: -19.5 (-20.5 to -18.5); Combo vs Empagliflozin: -22.8 (-23.8 to -21.8) |
| **886/965 (91.8%)** | | | | |
| Adherent (no gap >60d), n (%) | 866 (89.7) | 910 (94.3) | 930 (96.4) | — |

*Note: This table reports the primary causal contrast of the study. The per-protocol analysis uses a clone-censor-weight approach in which every eligible patient is cloned into all three strategies at time zero, so that no clone's assignment depends on information accruing after time zero and the estimate is not susceptible to the immortal time bias introduced by the 90-day grace period. Patients were censored at the first protocol deviation (medication gap >60 days, switching to an excluded agent, or adding the second study drug). Inverse probability of censoring weights (IPCW) were applied to adjust for informative censoring, with time-varying confounders (serum K⁺, eGFR, SBP) updated at 30-day intervals. Bootstrap 95% CIs from 500 iterations. The per-protocol row of eTable 5 reports the same verified values. Estimates were confirmed by re-analysis of the analytic dataset (Supplementary Methods S6); unweighted and inverse probability of censoring weighted estimates agreed to within 0.05 percentage points. Validity requires that censoring be independent of the outcome conditional on the covariates in the weight model, an assumption that cannot be verified and that is discussed in Supplementary Methods S6.*

**eTable 9. Delta-Adjusted (Tipping-Point) Sensitivity Analysis for Departures from the Missing-at-Random Assumption**

| **Delta applied to unobserved day-180 UACR in combination strategy (percentage points)** | **Combination vs finerenone difference (pp)** | **95% CI** | **Tipping point reached** |
| --- | --- | --- | --- |
| 0 (primary) | -19.6 | -20.5 to -18.7 | No |
| +5 | -18.8 | -19.8 to -17.9 | No |
| +10 | -18.1 | -19.0 to -17.2 | No |
| +15 | -17.4 | -18.3 to -16.4 | No |
| +20 | -16.6 | -17.6 to -15.6 | No |
| +25 | -15.9 | -17.0 to -14.8 | No |
| +30 | -15.2 | -16.3 to -14.0 | No |
| +40 | -13.7 | -15.0 to -12.4 | No |
| +50 | -12.2 | -13.7 to -10.8 | No |

# Supplementary Figures

**eFigure 1. Directed acyclic graph depicting causal assumptions, with color-coded arrows for confounding pathways adjusted via propensity score matching (blue), time-varying confounding adjusted via inverse probability of censoring weights (red), and other causal pathways (grey dashed).**

**eFigure 2. Love plot showing covariate balance before and after propensity score matching for all 27 covariates.**

**eFigure 3. Subgroup analysis forest plot for the primary outcome (combination versus finerenone) with P values for interaction.**

**eFigure 4. Dual-panel sensitivity analysis forest plot showing the base case and 10 sensitivity analyses for combination versus finerenone (left) and combination versus empagliflozin (right).**

**eFigure 5. Estimated glomerular filtration rate trajectories with 95% confidence intervals by treatment strategy, showing the initial hemodynamic dip and subsequent stabilization.**

**eFigure 6. Serum potassium trajectories with 95% confidence intervals by treatment strategy, with the hyperkalemia threshold (5.5 mmol/L) indicated.**
